# Populist attitudes and belief in conspiracy theories: anti-elitist attitudes and the preference for unrestricted popular sovereignty reduce the positive impact of an analytical thinking style on conspiracy beliefs

**DOI:** 10.1186/s13104-025-07136-z

**Published:** 2025-02-11

**Authors:** Stephanie Mehl, Winfried Rief, Daniel Soll, Nico Pytlik

**Affiliations:** 1 Department of Psychiatry and Psychotherapy & Center for Mind, Brain and Behavior (MCMBB), Philipps University, Rudolf-Bultmann-Str. 8, 35039 Marburg, Germany; 2 Department of Clinical Psychology and Psychotherapy, Philipps University, Gutenbergstraße 18, 35037 Marburg, Germany; 3 Department of Social Work and Health, University of Applied Sciences Frankfurt am Main, Nibelungenplatz 1, 60318 Frankfurt am Main, Germany

**Keywords:** Conspiracy beliefs, Populism, Anti-elitism, Preference for popular sovereignty, Analytical thinking style

## Abstract

**Objective:**

Populist attitudes and the tendency to believe in specific conspiracy theories (conspiracy beliefs) are often exploited by extremist or populist parties. However, more scientific research is needed to scrutinize this association. Consequently, the present non-preregistered exploratory online study assessed whether and how conspiracy beliefs and populist attitudes are associated and whether populist attitudes moderate the association between the preference for an analytical or intuitive thinking style and conspiracy beliefs.

**Results:**

We assessed 483 nonclinical individuals regarding their conspiracy beliefs, populist attitudes, and thinking styles and found a moderate correlation between populist attitudes and conspiracy beliefs. Conspiracy beliefs were significantly predicted by three facets of populist attitudes (anti-elitism, preference for unrestricted popular sovereignty, and belief in the homogeneity and virtuousness of the people). Anti-elitist attitudes and a preference for unrestricted popular sovereignty significantly moderated (reduced) the impact of an analytical thinking style on conspiracy beliefs. Anti-elitism and a preference for popular sovereignty might enhance a person’s vulnerability to conspiracy beliefs. We assume that these populistic attitudes reduce a person’s motivation to use a more effortful thinking style to reinterpret ideology-inconsistent information to protect existing conspiracy beliefs. Our results provide new insights into the interplay between conspiracy beliefs, populism, and a preference for an analytical/more effortful thinking style.

**Supplementary Information:**

The online version contains supplementary material available at 10.1186/s13104-025-07136-z.

## Introduction

*Beliefs in conspiracy theories/conspiracy beliefs* are defined as tendencies to explain the ultimate cause of significant social and political events through secret plots by two or more powerful people with sinister intentions [1–3]. In addition, a general tendency to endorse conspiracy beliefs is defined as a *conspiracy mentality* [4]. During the SARS-CoV-19 pandemic, conspiracy beliefs flourished due to uncertainty, fear, and complexity, and their supporters became the center of societal and political debates. Several new conspiracy beliefs have emerged regarding the intentional spread of the virus by sinister actors who harmed societies with sanitary measures and vaccines [5]. Conspiracy beliefs were found to have behavioral consequences for people’s health and well-being: several studies reported that persons who endorsed the pandemia-related conspiracy beliefs were less inclined to participate in countermeasures to prevent the spread of the virus (meta-analysis by [6]). Persons who endorsed pandemic-related conspiracy beliefs were less motivated to test or vaccinate themselves or adhere to government guidelines [7, 8] and showed less pronounced trust in health professionals and health institutions [9]. In addition, conspiracy beliefs also reduce a person’s motivation to engage in politics in normative ways (e.g., demonstrations, voting) [10, 11] and their pro-environmental behavior [8, 11], while increasing the motivation to employ illegal means of political articulation (e.g., illicit demonstrations, spreading incorrect information, hacking). Furthermore, conspiracy beliefs also increase prejudices such as Antisemitism, Islamophobia, and inter-group hostility [12, 13].

Social and traditional media are involved in spreading conspiracy beliefs, and the increase in social media use in recent years has ignited this development even more [14] by moderating (intensifying) the association between conspiracy beliefs and diminished trust in institutions [15].

Distrust in institutions is a prominent feature of populism. Populism is defined by Mansbridge and Macedo [16] in four categories: (1) the belief in a *homogenous group of people* seen as coherent entities of persons who are pure, good, honest, virtuous, and upright and are believed to be the only legitimate actors in a democracy [17, 18]; (2) *exclusive people*, a morally corrupt, powerful elite dominating ordinary people with specific financial interests that often includes pronounced anti-elitism [19]; (3) *greater direct popular rule* which refers to the suggestion that ordinary people are morally superior and should thus be involved in political decisions more directly; and (4) *nationalism*, the fight for a bounded political community of homogeneous people (whereas some populist movements are more globally focused). Populistic attitudes represent a dangerous threat to democratic processes that are based upon genuine disagreement, diversity of interest among heterogeneous groups, negotiation, and compromise [16].

Thus, populism has several similarities with conspiracy beliefs: Persons who endorse conspiracy beliefs also assume that an elite group colludes to gain personal advantages over the risks of adverse effects for ordinary people and simultaneously question establishments, institutions, media, and science [20]. However, there are also marked differences between the two distinct constructs: people who endorse conspiracy beliefs do not view themselves as part of the ordinary population but as part of an alternative elite group engaged in uncovering political and societal conspiracies [21]. Nevertheless, both constructs are based on a common personality disposition: generalized dispositional distrust [22].

Interestingly, in an online experiment, exposure to populistic information and conspiracy beliefs about the health system resulted in more pronounced populist attitudes than listening solely to populist attitudes or neutral information [23]. In addition, van Prooijen et al. [24] found that persons with politically extreme views were more prone to conspiracy beliefs (quadratic association) and that a pronounced belief in simple political solutions mediated this association. Thus, conspiracy beliefs are related to populism, political extremism, and religious fundamentalism and might even enhance the violent tendencies of extreme political groups [25]. Thus, efforts to manipulate the public via conspiracy beliefs and populistic content might harm a democracy [21].

Although several online studies assessing conspiracy beliefs in social media have been conducted in recent years (reviewed by Mahl et al. [26]), only a few studies have directly evaluated the relationship between conspiracy beliefs and populism. Castanho Silva et al. [27] found an association between populist attitudes and theories about malevolent groups controlling world events, whereas other conspiracy beliefs related to crime, terrorism, and organizations secretly harming people’s health were unrelated to populist attitudes. However, it would be interesting to know whether the specific core categories of populism proposed by [16], such as belief in a homogeneous group of people, anti-elitism, and preference for unrestricted popular sovereignty, are associated with conspiracy beliefs.

Recent research has revealed that conspiracy beliefs are associated with specific thinking styles. Several *dual-process models* of human reasoning distinguish between an *analytical thinking style* that requires cognitive effort and is therefore dependent on working memory and cognitive abilities and an *intuitive thinking style* that involves a faster and effortless route of reasoning relying on heuristics and intuition and is considered independent of working memory and cognitive skills [28, 29]. However, Newton and colleagues [30] criticized the assumption of two distinct thinking styles and found evidence for four distinct types of thinking styles: actively open-minded thinking, close-minded thinking, preference for intuitive thinking, and preference for effortful thinking.

Interestingly, a preference for a more analytical or effortful thinking style protects people from endorsing conspiracy beliefs. In a meta-analysis, an analytical thinking style was associated with less pronounced beliefs in conspiracy theories [31]. Ståhl and van Prooijen [32] even found that a preference for analytical thinking was associated with conspiracy beliefs (and paranormal phenomena) only in persons less motivated to base their beliefs on rational grounds (epistemic rationality).

However, interventions that induce an analytical thinking style do not always protect individuals from conspiracy beliefs. O’Mahony and colleagues [33] investigated the effectiveness of several interventions to reduce conspiracy beliefs and found that most interventions were ineffective in changing these beliefs. However, interventions focused on detecting human errors in perception by applying logic and critical scientific thinking skills showed some promising results [34].

In addition, a person’s preexisting opinions, evaluations, and conspiracy beliefs should be considered when planning interventions to reduce conspiracy beliefs. This assumption is supported by [35], who asked people to analyze scientific data on skin rashes (neutral condition) and gun control. Data on gun control was presented in either an ideology-consistent or inconsistent way. They found that persons with more pronounced cognitive abilities were more prone to misinterpreting data that contrasted with their political views (data in the ideology-inconsistent condition).

Thus, it can be assumed that strong underlying political views, such as populist attitudes, may influence analytic thinking abilities and reduce the preventive impact of an analytical thinking style on beliefs in conspiracy theories. This assumption aligns with the two-component socio-epistemic model of belief in conspiracy theories [36], which assumes that conspiracy theories as expressions of ‘epistemic mistrust’ are closely related to the processing of misinformation and several other individual psychological and societal processes, e.g., lack of analytical thinking, mistrust, threat, paranoia, and cognitive biases. In conclusion, it can be assumed that populistic attitudes might moderate (reduce) the positive impact of analytical thinking on conspiracy beliefs. Anti-elitism is particularly interesting, as it shows the most pronounced association of all populism facets with conspiracy beliefs [27]. In addition, it would be intriguing to test whether populist attitudes moderate the positive impact of an intuitive thinking style on conspiracy beliefs.

In conclusion, the first exploratory research question of the present non-preregistered online study assesses whether the endorsement of conspiracy beliefs and generalist populist attitudes are associated. The second exploratory research question tests whether the three core categories of populist attitudes (anti-elitism attitudes, attitudes assuming a homogenous and virtuous group of people, and a preference for unrestricted popular sovereignty) and conspiracy beliefs are related. The third exploratory research question assesses whether the three categories of populist attitudes moderate the association between the preference for an analytical or intuitive thinking style and conspiracy beliefs.

## Methods

Participants in the present non-preregistered exploratory cross-sectional online study were recruited via social media (Facebook) and a scientific survey-sharing platform (Survey Circle [37]) that allows scientists to publish surveys and recruit participants in exchange for participation in other online surveys. In addition, participants were offered to take part in a voluntary lottery for 15 Amazon vouchers (20 € each) as an additional incentive.

Participants were included if they were older than 16 years, had internet and social media access, and if they or their legal guardian signed an informed consent form. They were informed that the study investigated associations between political attitudes and decision behavior and were then asked to provide their consent.

First, patients’ tendency to jump to conclusions was examined using the fish task (the results have been reported [38]). The participants subsequently answered a self-designed questionnaire on conspiracy beliefs (see Supplement, Tables S2, and S3), the *Populist Attitudes Questionnaire* [18], which assesses populist attitudes, the *Rational-Experiential Inventory* [39], which measures the preference for an analytical or intuitive thinking style, and a sociodemographic questionnaire (a more detailed description of the measures can be found in Table S1). Finally, the participants were asked whether they had answered all the questions conscientiously and truthfully, whether they considered their data valid, and whether they recommended data use. The formal ethical review/approval requirement was waived by the University of Marburg’s Ethics Committee (Faculty of Psychology), as no experimental manipulation occurred, participants received information about the study, provided written informed consent, and were assured anonymity. The study was conducted in accordance with local legislation and institutional requirements. All study materials can be viewed on the Open Science Foundation’s project page (https://osf.io/bkv2p). Statistical analyses were performed using SPSS version 29 and the PROCESS macro [40], and are described in more detail in Table S4.

## Results

### Sample characteristics

Among the original 533 participants, 519 signed the informed consent form. In addition, 7 participants were excluded because they declared their data were invalid. One subject who reported suffering from schizophrenia in the past was excluded because paranoid ideation might influence conspiracy beliefs [41, 42].

The mean time for completion in the sample (*n* = 511) was 866.18 s (standard deviation (*SD*) = 316.18; range: 95-2225). To prevent data fraud that might be expected on a platform recommending that researchers participate in other studies to improve their study recruitment process, we excluded all participants with a substantially lower completion time than others (z equal to or smaller than − 1.96; *n* = 23), as recommended by [43], to improve the results of online studies. In the reduced sample (*n* = 488), the mean completion time was 899.39 s (*SD* = 282.88; range: 263–2225). For the main analyses, we excluded persons who reported being assigned at birth to diverse sex (*n* = 5) but included them in additional analyses. This decision resulted in a final sample size of 483 participants and a mean completion time in the final sample of 896.84 s (SD = 282.05; range: 263–2225).

The education level in our sample is notably high, with 60% of participants reporting a university degree (Table S5 and Table S31). Group differences between persons who reported being assigned at birth as male or female were examined via univariate ANOVAs (see Table S6). There were no statistically significant group differences in the endorsement of conspiracy beliefs (Conspiracy Beliefs Scale (CB)) and anti-elitist attitudes (Anti-Elitist Attitudes Scale (Anti)). However, women showed more pronounced general populist attitudes (Generalist Populist Attitudes Scale (Gen)), a more pronounced preference for unrestricted popular sovereignty (Preference for Unrestricted Popular Sovereignty Scale (Sov)), more pronounced beliefs in the homogeneity and virtuousness of the people (Beliefs in the Homogeneity and Virtuousness of the People Scale (Hom)) and a more pronounced preference for an intuitive thinking style (Faith in Intuition Scale (FI)) than men. Men presented a more pronounced preference for analytical thinking style (Need for Cognition Scale (NC)). Analyses were repeated, including persons of diverse sex, with mostly comparable results (see Table S32).

As shown in Table S7, endorsement of conspiracy beliefs (CBs) and a preference for analytical thinking (NC) were positively associated with age. In contrast, a preference for intuitive thinking (FI) was negatively correlated with age. Endorsement of conspiracy beliefs (CBs), all facets of populist attitudes (Gen, Anti, Sov, and Hom), and a preference for an intuitive thinking style (FI) were negatively associated with education level.

### Correlation between conspiracy beliefs and general populist attitudes (Research question 1)

The means and standard deviations of the individual conspiracy beliefs are depicted in Table S28. The results of a partial correlation analysis, controlling for age, education level, and sex assigned at birth, revealed a statistically significant correlation between conspiracy beliefs (CB) and more pronounced general populist attitudes (Gen: *r* (474) = 0.404, *p* <.001, 95% CI [0.32; 0.48]). Analyses were repeated, including persons with diverse sex assigned at birth (*n* = 5), and the results were comparable (*r* (478) = 0.403, *p* <.001, 95% CI [0.33; 0.49])). Table 1 provides an overview of the partial correlation coefficients between CB, populist attitudes (Gen, Anti, Hom & Sov), and thinking style subscales (NC, FI), controlling for age, education level, and sex assigned at birth. The results revealed statistically significant positive correlations between conspiracy beliefs (CB), populist attitudes (Gen, Anti, Hom, Sov), and a preference for an intuitive thinking style (FI). The preference for an analytical thinking style (NC) was negatively associated with all other measures (CB, Gen, Anti, Hom, Sov, and FI). The results were comparable in a partial correlation analysis, including persons with diverse sex assigned at birth (Table S8), and in a correlation analysis without controlling for covariates (Table S9).

**Table 1:** Partial correlations between conspiracy belief, populist attitudes, and analytical and intuitive thinking styles controlling for age, education level, and sex assigned at birth

	CB	Gen	Anti	Sov	Hom	NC	FI
Endorsement of Conspiracy Belief Scale (CB)	1						
Generalist Populist Attitudes Scale (Gen)	404**^1^	1					
Anti-Elitist Attitudes Scale (Anti)	0.373**^1^	0.761**^2^	1				
Preference for Unrestricted Popular Sovereignty Scale (Sov)	0.309**^1^	0.789**^2^	0.535**^2^	1			
Belief in the Homogeneity and Virtuousness of the People Scale (Hom)	0.218**^1^	0.668**^2^	0.176**^2^	0.250**^2^	1		
Need for Cognition Scale (NC)	− 0.175**^1^	− 0.346**^2^	− 0.241**^2^	− 0.199**^2^	− 0.318**^2^	1	
Faith in Intuition Scale (FI)	0.360**^1^	0.347**^2^	0.272**^2^	0.246**^2^	0.250**^2^	− 0.323**^2^	1

Notes: ^**^: *p* <.001; ^1^*n* = 479; ^2^*n* = 483; CB = Endorsement of Conspiracy Beliefs Scale; Gen = Generalist Populist Attitudes Scale; Anti = Anti-elitist Attitudes Scale; Sov = Preference for Unrestricted Popular Sovereignty Scale; Hom = Belief in the Homogeneity and Virtuousness of the People Scale; NC = Need for Cognition Scale; FI = Faith in Intuition Scale

### Associations between conspiracy beliefs and different facets of populist attitudes

A hierarchical linear regression analysis was performed, controlling for age, education level, and sex assigned at birth. The results are depicted in Table 2.

**Table 2:** Linear regression analysis of the ability of populism to predict conspiracy beliefs controlling for age, education level, and sex assigned at birth

		Unstandardized coefficients		Standardized coefficients									
Step	Predictor	*B*	*SE*	*Beta*	*p*			*R* ^*2*^		*R* ^*2 change*^	*F/F* ^*change*^ *(df1/df2)*		*p*
1								0.043		0.043	*F* (3,475) = 7.171		< 0.001
	Age	0.011	0.004	0.113	0.013								
	Sex assigned at birth	0.136	0.070	0.088	0.053								
	Education level	− 0.142	0.041	− 0.154	< 0.001								
2								0.209		0.166	*F* (3,472) = 32.991		< 0.001
	Age	0.009	0.004	0.092	0.027								
	Sex assigned at birth	0.089	0.065	0.057	0.173								
	Education level	− 0.058	0.039	− 0.063	0.134								
	Anti	0.294	0.052	0.282	< 0.001								
	Sov	0.106	0.044	0.122	0.015								
	Hom	0.156	0.050	0.137	0.002								

Notes: SE = standardized error of B; Anti = Anti-elitist Attitudes Scale; Sov = Preference for Unrestricted Popular Sovereignty Scale; Hom = Belief in the Homogeneity and Virtuousness of the People Scale-

The model explained 4.3% of the total variance in conspiracy belief (CB). Including the three populist subscales (Anti, Hom & Sov) in the second step significantly improved the model. The model explained 21% of the variance in conspiracy beliefs (CB), and age, anti-elitist attitudes (Anti), demand for unrestricted popular sovereignty (Sov), and belief in the homogeneity and virtuousness of the people (Hom) were statistically significant predictors. Table S10 depicts the regression analysis without controlling for covariates. The model explained 20.9% of the variance in conspiracy beliefs and age, and the three facets of populism (Anti, Sov, & Hom) were statistically significant predictors. The moderation analysis was repeated including persons with diverse sex assigned at birth with similar results (male sex assigned at birth was an additional significant predictor, see Table S10). Table S11 shows the moderation analysis without controlling for covariates with comparable results.

### Moderation analysis: do populist attitudes moderate the association between a preference for an analytical thinking style and endorsement of conspiracy beliefs

Table 3 depicts the results of the moderation analysis (hierarchical linear regression analysis) that assessed whether anti-elitist attitudes (Anti) moderate the association between a preference for analytical thinking style (NC) and the endorsement of conspiracy beliefs (CB). The first regression model was statistically significant and explained 18.4% of the variance in CB. The second model, which added the interaction term (Anti x NC), significantly explained an additional 1.7% of the variance in CB, bringing the total variance to 20.0%.

**Table 3:** Moderator analysis (hierarchical linear regression analysis) on whether anti-elitist attitudes moderate the association between a preference for analytical thinking and endorsement of conspiracy beliefs, controlling for age, sex assigned at birth, and education level

		Unstandardized coefficients			Standardized coefficients					
Step	Predictor	*B*	*SE*		*Beta*	*p*	*R* ^*2*^	*R* ^*2 change*^	*F/F* ^*change*^ *(df1/df2)*	*p*
1							0.184	0.184	*F* (5, 473) = 21.267	< 0.001
	Age	0.010	0.004		0.101	0.018				
	Sex assigned at birth	0.108	0.066		0.070	0.102				
	Education level	− 0.078	0.039		− 0.085	0.046				
	NC	− 0.074	0.036		− 0.090	0.041				
	Anti	0.365	0.046		0.350	< 0.001				
2							0.200	0.017	*F* (1, 472) = 9.857	0.002
	Age	0.010	0.004		0.099	0.019				
	Sex assigned at birth	0.094	0.066		0.060	0.154				
	Education level	− 0.069	0.039		− 0.074	0.077				
	NC	− 0.093	0.036		− 0.114	0.010				
	Anti	0.209	0.067		0.201	0.002				
	Interaction Anti x NC	0.141	0.045		0.196	0.002				

Notes: SE = standardized error of B; Anti = Anti-elitist Attitudes Scale; NC = Need for Cognition Scale

**Fig. 1:**
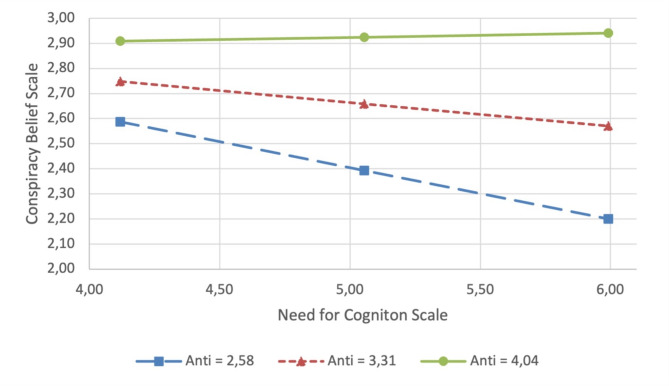
Results of the moderator analysis: Anti-elitist attitudes moderate the association between a preference for an analytical thinking style and endorsement of conspiracy beliefs. Note: Anti = Anti-elitist Attitudes Scale; Low scores in the Anti-Elitist Attitudes Scale = 2.58; Average scores in the Anti-elitist Attitudes Scale = 3,31; High scores in the Anti-elitist Attitudes Scale = 4.04

More specifically, the Johnson‒Neyman boundary of significance showed that this effect was statistically significant (*p* <.01) for anti-scores between 0 and 3.47. These findings suggest that lower levels of anti-elitist attitudes moderate (reduce) the positive impact of a preference for an analytical thinking style (NC) on the endorsement of conspiracy beliefs (CB). Figure 1 graphically represents the statistically significant moderation. The moderation analysis was repeated, including persons with diverse sex assigned at birth (Table S12) and without controlling for covariates (Table S13), and these analyses revealed comparable results.

The results of a moderation analysis (hierarchical linear regression analysis) assessing whether beliefs in the homogeneity and virtuousness of the people (Hom) moderate the association between a preference for an analytical thinking style (NC) and conspiracy beliefs (CB) are depicted in Table S14. The first regression model explained 10.7% of the variance in CB. The second model, including the interaction term (Hom x NC), did not explain an additional amount of variance in CB. Moderation analyses were repeated, including persons with diverse sex assigned at birth (Table S15) and without controlling for covariates (Table S16), and the results were comparable. These findings indicate that beliefs in the homogeneity and virtuousness of the people (Hom) were not found to moderate the association between a preference for analytical thinking (NC) and endorsement of conspiracy beliefs.

Regarding the question of whether a preference for unrestricted popular sovereignty (Sov) moderates the association between a preference for an analytical thinking style (NC) and conspiracy beliefs (CB), the first model explained 14.7% of the variance in CB. The second model, which added the interaction term (Sov x NC), explained an additional 1% of the variance in CB, significantly increasing the total variance to 15.6% (Table 4).

**Table 4:** Moderator analysis (hierarchical linear regression analysis) on whether a preference for unrestricted popular sovereignty moderates the association between a preference for an analytical thinking style and endorsement of conspiracy beliefs, controlling for age, education level, and sex assigned at birth

		Unstandardized coefficients			Standardized coefficients					
Step	Predictor	*B*	*SE*		*Beta*	*p*	*R* ^*2*^	*R* ^*2 change*^	*F/F* ^*change*^ *(df1/df2)*	*p*
1							0.147	0.147	*F* (5, 473) = 16.326	< 0.001
	Age	0.011	0.004		0.118	0.007				
	Sex assigned at birth	0.051	0.068		0.033	0.453				
	Education level	− 0.094	0.040		− 0.102	0.019				
	NC	− 0.097	0.036		− 0.119	0.008				
	Sov	0.247	0.038		0.284	< 0.001				
2							0.156	0.009	*F* (1, 472) = 5.012	0.026
	Age	0.011	0.004		0.116	0.007				
	Sex assigned at birth	0.045	0.067		0.029	0.506				
	Education level	− 0.083	0.040		− 0.091	0.037				
	NC	− 0.113	0.037		− 0.138	0.002				
	Sov	0.145	0.059		0.167	0.015				
	Interaction NC x Sov	0.086	0.038		0.150	0.026				

Notes: SE = standardized error of B; NC = Need for Cognition Scale; Sov = Preference for Unrestricted Popular Sovereignty Scale

**Fig. 2:**
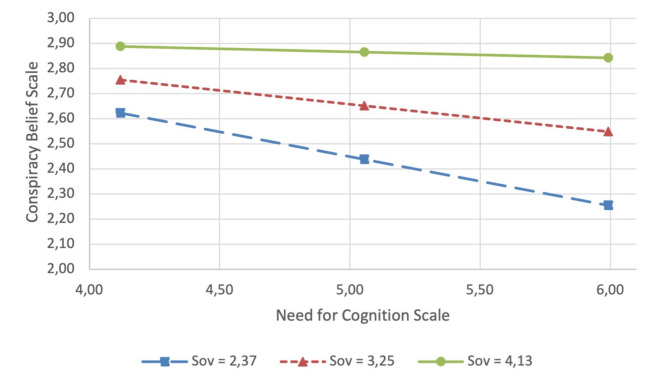
Results of the moderator analysis: does a preference for unrestricted popular sovereignty moderate the association between a preference for an analytical thinking style and endorsement of conspiracy beliefs? *Note*: Sov = Preference for Unrestricted Popular Sovereignty Scale; Low scores in the Preference for Unrestricted Popular Sovereignty Scale = 2.37; Average scores in the Preference for Unrestricted Popular Sovereignty Scale = 3.25; High scores in the Anti-elitist Attitudes Scale = 4.13

The Johnson‒Neyman boundary of significance shows that this effect was statistically significant (*p* <.01) for Sov mean scores between 0 and 3.71. These findings suggest that lower levels of a preference for unrestricted popular sovereignty (Sov) moderate (reduce) the positive impact of a preference for analytical thinking (NC) on the endorsement of conspiracy beliefs (CB). A graphic representation of the moderation is shown in Fig. 2. The moderation analysis was repeated, including persons with diverse sex assigned at birth (Table S17) and without controlling for covariates (Table S18), and these analyses revealed comparable results.

### Moderator analysis: do populist attitudes moderate the association between a preference for an intuitive thinking style and endorsement of conspiracy beliefs

Three moderation analyses (hierarchical linear regression analyses) were performed to assess whether the three populist attitudes (Anti, Hom, Sov) moderate the association between a preference for an intuitive thinking style (FI) and endorsement of conspiracy beliefs (CB). Results for the moderation analyses controlling for age, education level, and sex assigned at birth are depicted in Table S19, Table S22, and Table S25. In all moderation analyses, the second model, including the interaction term (Anti/Hom/Sov x FI), did not explain an additional amount of variance in CB. Analyses were repeated without controlling for covariates (Table S21, Table S24, and Table S27) and including persons of diverse sex (Table S20, Table S23, and Table S26), and all moderation analyses were not statistically significant.

## Discussion

The present study revealed a moderate correlation between conspiracy beliefs and general populist attitudes. More specifically, all three dimensions of populist attitudes (anti-elitist attitudes, preference for unrestricted popular sovereignty, and a belief in the homogeneity and virtuousness of the people) predicted the endorsement of conspiracy beliefs. Furthermore, anti-elitist attitudes moderated the association between the preference for an analytic or more effortful thinking style and conspiracy beliefs: the more pronounced a person’s anti-elitist attitudes are, the less a preference for an analytical and more effortful thinking style counteracts the endorsement of conspiracy beliefs. Additionally, the preference for unrestricted popular sovereignty also moderated (reduced) the association between the preference for an analytical and more effortful thinking style and the endorsement of conspiracy beliefs.

Our findings align with the close connection between populism and conspiracy beliefs described in the scientific literature [44, 45]. In an online study [46] that used the same questionnaire for populist attitudes, a strong association between populist attitudes and conspiracy beliefs could also be detected: anti-elitism, demand for unrestricted sovereignty, and belief in the homogeneity of the people were associated with five facets of conspiracy beliefs with medium to large correlation coefficients (*r* =.26 −.68) that are comparable to the correlation coefficients we found (*r* =.19 −.79: see Table S31). In addition, our results align with the results of the online study by [27], who reported an association between populism and specific conspiracy beliefs regarding malevolent groups controlling world events. More generally, our results also align with the quadratic association between conspiracy beliefs in persons with extreme political views (political left-wing and right-wing) [24]. The finding that the endorsement of conspiracy beliefs is closely related to different kinds of populist attitudes can be explained by Thielmann and Hilbig [22], who identified a personality factor as the common core of the endorsement of conspiracy beliefs and populist attitudes: generalized dispositional distrust.

Regarding the effect of both anti-elitist attitudes and the preference for unrestricted sovereignty in moderating (reducing) the positive influence of a preference for analytical thinking on endorsements of conspiracy beliefs, these results must be viewed cautiously as our measure of a preference for analytical and intuitive thinking styles, the Rational-Experiential Inventory [47], might suggest that analytic and intuitive thinking styles are fundamentally distinct endpoints on one spectrum of thinking styles. However, Newton and colleagues [30] reported evidence that classifying persons as either “intuitive” or “analytical” is an oversimplification, and they suggested four different intuitive-analytical thinking styles: actively open-minded thinking, close-minded thinking, a preference for intuitive thinking, and a preference for effortful thinking. Thus, we solely assessed the moderation of populist attitudes on the association between two intuitive-analytical thinking styles and conspiracy beliefs. We might have underestimated the role of other thinking styles. In addition, our measure of analytical thinking should instead be named a preference for effortful thinking, as suggested by [30].

However, our findings suggest that persons with lower anti-elitist attitudes and beliefs in a more direct popular rule might be more motivated to use their analytical thinking and reasoning styles to critically test the veracity of conspiracy beliefs, resulting in less pronounced endorsement of these beliefs. However, in persons with more pronounced populist attitudes, these attitudes might supersede an individual’s tendency to use their analytic skills to critically assess conspiracy beliefs and consider alternative explanations and interpretations. These individuals might instead engage in motivated reasoning [48] in favor of the assumptions of conspiracy beliefs. In addition, they might be more likely to fall prey to cognitive biases that could impact their analytical thinking skills (e.g., reasoning biases [49] and confirmation bias [50]). This tendency might be driven by a pronounced need for self-deception and closure [51] and may be more pronounced in persons less motivated by epistemic rationality [32]. The fact that populist attitudes did not moderate (enhance) the impact of a preference for an intuitive thinking style on conspiracy theories corroborates this assumption.

These assumptions need to be tested in further experimental studies. Overall, other existing ideological perspectives may also diminish the positive impact of analytical thinking on reducing conspiracy beliefs by misinterpreting contradictory evidence, which is then integrated into the existing conspiracy belief system.

### Limitations

Several limitations need to be acknowledged. We used the traditional variant of measuring conspiracy beliefs by asking the subjects to give their approval regarding several specific conspiracy beliefs. This approach hinders direct replication of the study, as the prevalence and approval of particular conspiracy beliefs vary across (sub)cultures and times [52]. We recruited the participants via two platforms (Facebook and Survey Circle) and did not ask them to report how they became aware of our study. Thus, we cannot compare both groups regarding sociodemographic variables or other measures. Recruiting participants via the Survey Circle platform might not be ideal, as participants were not implicitly motivated to participate in our online studies but joined in our study to improve the recruitment of their online studies. In addition, our findings are based on a sample that can be considered a convenience sample.

Regarding sociodemographic variables, we asked participants to report their sex assigned at birth. We offered the option “diverse sex” but were only able to recruit a small number of participants (*n* = 5). Although our sample is comparable in terms of the ratio of assigned sex at birth with a representative sample [53], our sample is nevertheless considerably younger and better educated. It can be considered as weird (*w*hite, *E*uropean, *i*ndustrialized, *r*ich, and *d*emocratic) [54], which has been generally criticized in conspiracy belief research [26]. Moreover, as mentioned above, by using the Rational-Experiential Inventory [47], we could not assess the four distinct analytical-intuitive thinking styles identified by [30]. Finally, our study was not pre-registered (while we shared all the data and analyses).

## Conclusion

In conclusion, we were able to shed light on the theoretically well-established association between conspiracy beliefs and populist attitudes. We found evidence of an association between conspiracy beliefs and populism. Anti-elitist attitudes and a preference for direct popular sovereignty, as core characteristics of populism, seem to play essential roles in the formation of conspiracy beliefs and appear to moderate (reduce) the positive influence of a preference for a more effortful thinking style on conspiracy beliefs. Understanding the causes of conspiracy beliefs, populist attitudes, and the interrelation of both could be essential not only for strengthening the democratic discourse but also for reducing the social rifts that have opened up in recent years.

## Electronic supplementary material

Below is the link to the electronic supplementary material.

Supplementary Material 1

## Data Availability

All the stimuli, presentation materials, and participant data are available on the OSF’s project page: https://osf.io/bkv2p.
