# Supplementary material for: Populist attitudes and belief in conspiracy theories: anti-elitist attitudes and the preference for unrestricted popular sovereignty reduce the positive impact of an analytical thinking style on conspiracy beliefs
Source: BMC Res Notes. 2025 Feb 11;18:63. doi: 10.1186/s13104-025-07136-z (PMC11817401; doi:10.1186/s13104-025-07136-z)
Supplement: Supplementary file 1 — Supplementary Material 1 [file 13104_2025_7136_MOESM1_ESM.docx]

Supplementary Material

**Populist attitudes and belief in conspiracy theories: Anti-elitist attitudes and the preference for unrestricted popular sovereignty reduce the positive impact of an analytical thinking style on conspiracy beliefs**

Stephanie Mehl^1,2^, Winfried Rief ^3,^ Daniel Soll ^2^, Nico Pytlik^3^

^1^ Department of Health and Social Work, University of Applied Sciences Frankfurt, Frankfurt am Main, Germany

^2^ Department of Psychiatry and Psychotherapy & Center for Mind, Brain and Behavior (MCMBB), Philipps-University, Marburg, Germany

^3^ Department of Clinical Psychology and Psychotherapy Philipps-University, Marburg, Germany

***** Corresponding Author: Stephanie.mehl@staff.uni-marburg.de

This supplement contains supporting information for the manuscript mentioned above. The results of complementary analyses are reported, and the findings are discussed in the main document.

**Table S1:** Measures 5

**Table S2:** Original German conspiracy belief items 6

**Table S3:** English translation of conspiracy belief items 11

**Table S4:** Statistical analyses 10

**Table S5:** Sociodemographic characteristics 12

**Table S6:** Group differences in groups regarding sex assigned at birth in endorsement of 14

conspiracy beliefs, populist attitudes, and analytical and intuitive thinking style

**Table S7:** Correlations between age, education level, and endorsement of conspiracy 15

beliefs, populist attitudes, and analytical and intuitive thinking styles

**Table S8:** Correlations between endorsement of conspiracy beliefs, populist attitudes, 16

and analytical and intuitive thinking styles controlling for age, education level

and sex assigned at birth, including persons with diverse sex assigned at birth

**Table S9:** Correlations between endorsement of conspiracy beliefs, populist attitudes, 17

and analytical and intuitive thinking styles without controlling for covariates

**Table S10:** Linear regression analysis predicting endorsement of conspiracy belief 18

controlling for age, education level, and sex assigned at birth, including

persons with diverse sex

**Table S11:** Linear regression analysis on populistic attitudes predicting endorsement 19

of conspiracy beliefs without controlling for covariates

**Table S12:** Moderator analysis (hierarchical linear regression analysis) assessing whether 20

anti-elitist attitudes moderate the association between a preference for an

analytical thinking style and endorsement of conspiracy beliefs, including

persons with diverse sex

**Table S13**: Moderator analysis (hierarchical linear regression analysis) assessing whether 21

anti-elitist attitudes moderate the association between a preference for an

analytical thinking style and endorsement of conspiracy beliefs without controlling for covariates

**Table S14:** Moderator analysis (hierarchical linear regression analysis) assessing whether 22

the belief in the homogeneity and virtuousness of the people moderates the

association between a preference for an analytical thinking style and

endorsement of conspiracy beliefs, controlling for age, education level, and

sex assigned at birth

**Table S15:** Moderator analysis (hierarchical linear regression analysis) assessing whether 23

beliefs in the homogeneity and virtuousness of the people moderate the

association between a preference for an analytical thinking style and endorsement of conspiracy beliefs, controlling for age, education level, and sex assigned at

birth, including persons with diverse sex assigned at birth

**Table S16**: Moderator analysis (hierarchical linear regression analysis) assessing whether 24

beliefs in the homogeneity and virtuousness of the people moderate

the association between a preference for an analytical thinking style and

endorsement of conspiracy beliefs without controlling for covariates

**Table S17:** Moderator analysis (hierarchical linear regression analysis) assessing whether 25

a preference for unrestricted popular sovereignty moderates the association

between a preference for an analytical thinking style and endorsement of

conspiracy beliefs, controlling for age, education level, and sex assigned at

birth and including persons with diverse sex assigned at birth

**Table S18:** Moderator analysis (hierarchical linear regression analysis) assessing 26

whether the preference for unrestricted popular sovereignty moderates

the association between a preference for an analytical thinking style and

endorsement of conspiracy beliefs without controlling for covariates

**Table S19:** Moderator analysis (hierarchical linear regression analysis) assessing whether 27

anti-elitist attitudes moderate the association between a preference for an

intuitive thinking style and endorsement of conspiracy beliefs, controlling for age,

education level and sex assigned at birth

**Table S20:** Moderator analysis (hierarchical linear regression analysis) assessing whether 28

anti-elitist attitudes moderate the association between a preference for an

intuitive thinking style and endorsement of conspiracy beliefs, controlling

for age, education level, and sex assigned at birth, including persons with

diverse sex assigned at birth

**Table S21:** Moderator analysis (hierarchical linear regression analysis) assessing whether 29

anti-elitist attitudes moderate the association between a preference for intuitive

thinking and endorsement of conspiracy beliefs without controlling for covariates

**Table S22:** Moderator analysis (hierarchical linear regression analysis) on whether beliefs 30

in the homogeneity and virtuousness of the people moderate the association

between a preference for intuitive thinking and endorsement of conspiracy beliefs, controlling for age, education level, and sex assigned at birth

**Table S23**: Moderator analysis (hierarchical linear regression analysis) assessing whether 31

beliefs in the homogeneity and virtuousness of the people moderate the

association between a preference for intuitive thinking and endorsement of

conspiracy beliefs, controlling for age, education level, and sex assigned at birth

and including persons with diverse sex assigned at birth

**Table S24**: Moderator analysis (hierarchical linear regression analysis) assessing whether 32

beliefs in the homogeneity and virtuousness of the people moderate the

association between a preference for an intuitive thinking style and

endorsement of conspiracy beliefs without controlling for covariates

**Table S25:** Moderator analysis (hierarchical linear regression analysis) on whether a 33

preference for unrestricted popular sovereignty moderates the association

between a preference for intuitive thinking and endorsement of conspiracy

beliefs, controlling for age, education level, and sex assigned at birth

**Table S26:** Moderator analysis (hierarchical linear regression analysis) on whether the 34

preference for unrestricted popular sovereignty moderates the association

between a preference for an intuitive thinking style and endorsement of

conspiracy beliefs, controlling for age, education level, and sex assigned at

birth and including persons with diverse sex assigned at birth

**Table S27:** Moderator analysis (hierarchical linear regression analysis) on whether the 35

preference for unrestricted popular sovereignty moderates the association

between a preference for an intuitive thinking style and endorsement of conspiracy

beliefs, without controlling for covariates

**Table S28** Means and standard deviations of endorsed conspiracy beliefs 36

**Table S29:** Intercorrelations between individual endorsed conspiracy beliefs and populism 37

**Table S30:** Intercorrelations between individual endorsed conspiracy beliefs 38

**Table S31:** Sociodemographic characteristics in the sample, including persons 39

with diverse sex

**Table S32:** Group differences in groups regarding sex assigned at birth in endorsement 41

of conspiracy beliefs, populist attitudes, and analytical and intuitive thinking style

including persons with diverse sex assigned at birth

**Table S33:** Correlations between age, education level, and endorsement of conspiracy 42

beliefs, populist attitudes, and analytical and intuitive thinking styles, including

persons with diverse sex assigned at birth

**References**: 43

**Table S1: Measures**

*Conspiracy beliefs*

In the present study, conspiracy beliefs were measured by asking participants to rate several specific conspiracy beliefs regarding their respective approval (Brotherton et al., 2013). The mean approval rate is then interpreted as a measure of conspiracy beliefs. The different conspiracy beliefs were derived from a study that assessed the popularity and approval of 30 conspiracy beliefs in German-speaking countries (Bartoschek, 2015). We tested these 30 German conspiracy beliefs in a pilot study (Pytlik, 2016) and selected 20 beliefs with the highest discriminability for the present study. Participants were asked to read the conspiracy beliefs and then to rate their respective approval on 5-point Likert scales ranging from 1 (“I do not agree at all”) to 5 (“I fully agree”). If participants were unaware of the belief, they were asked to select the answer “Not able to judge/theory not known.” In this case, the rating was labeled missing and not included in the mean CB score. The mean approval rate of all judged/known conspiracy beliefs (*Conspiracy Beliefs Scale*) was used (range: 1-5). The final questionnaire had excellent reliability, with a Cronbach’s α of .94. The specific conspiracy beliefs are shown in Tables S2 and S3, and mean and standard deviations and intercorrelations between the individual CBs in Tables S28 and S30.

*Populist attitudes questionnaire*

Populist attitudes were measured using the three-dimensional German *Populist Attitudes Questionnaire* developed by Schulz, Schemer, Wirz, Wettstein & Wirth (2017), which consists of 15 items that are answered on 5-point Likert scales ranging from 1 (“strongly disagree”) to 5 (“strongly agree”). Following the conceptualization of Mudde (Mudde, 2004), the authors proposed and subsequently (factor-analytically) confirmed populist attitudes as a latent second-order construct based on three lower-order dimensions: (1) anti-elitist attitudes, (2) a preference for unrestricted popular sovereignty, and (3) belief in the homogeneity and virtuousness of the people. These dimensions are reflected in three subscales: *Anti-elitist Attitudes Scale* (Anti: mean score of 5 items), (2) *Preference of Unrestricted Popular Sovereignty Scale* (Sov: mean score of 4 items, range: 1-5), and (3) *Belief in the Homogeneity and Virtuousness of the People Scale* (Hom: mean score of 6 items, range: 1-5). In addition, the *Generalist Populist Attitudes Scale* (Gen: mean score of all items, range 1-5) assesses the general tendency to endorse populist attitudes. All scales presented good reliability (Anti: Cronbach’s α = .86; Sov: α = .86; Hom: α = .84), and the factorial structure was confirmed in two independent samples (Schulz et al., 2017).

*Analytical thinking style and intuitive thinking style*

Thinking preferences were assessed using the German version of the *Rational-Experiential Inventory* (Keller et al., 2000), which consists of 29 items answered on 7-point Likert scales ranging from 1 (“completely wrong”) to 7 (“completely true”). The original version proposed by Epstein, Pacini, Denes-Raj, and Heier (1996) is theoretically based on Epstein’s Cognitive-Experiential Self-Theory of Personality (2003). The questionnaire consists of 29 items measuring the individual’s thinking preferences using two subscales: 1) a dispositional preference for analytical thinking (Need for Cognition (Cacioppo & Petty, 1982) and 2) a preference for an intuitive thinking style. The *Need for Cognition Scale* (NC) consists of the mean score of 14 items (range: 1-7), and the *Faith in Intuition Scale* (FI) consists of the mean score of 15 items (range: 1-7). The subscales of the German version used in the present study feature good reliability, with Cronbach’s α = .82 (NC) and α = .86 (FI).

*Sociodemographic variables and additional measures*

The sociodemographic variables of the participants were also assessed: age, sex assigned at birth (male, female, or diverse), education level, identification with a religious group, religiosity, preferred German political party in a subsequent Bundestag election, and whether they had been diagnosed with a mental disorder in their past. Participants were assessed for jumping-to-conclusions bias using the fish task (Woodward et al., 2009).

**S2: Original German conspiracy beliefs items**

Bitte geben Sie nun an, inwiefern Sie den folgenden Verschwörungstheorien zustimmen. Mit dem Begriff Verschwörungstheorie soll dabei keinerlei Aussage, über die Richtigkeit einer Theorie getroffen werden. Diese Einordnung bedeutet lediglich, dass die Theorie der offiziellen Schilderung der Ereignisse widerspricht.

|  | Ich denke,… | Stimme gar nicht zu | Stimme eher nicht zu | Unent-schieden | Stimme eher zu | Stimme voll zu | **Kann ich nicht beurteilen/Theorie nicht bekannt** |
| --- | --- | --- | --- | --- | --- | --- | --- |
| 1 | … J. F. Kennedy wurde nicht von Lee Harvey Oswald (allein) erschossen. |  |  |  |  |  |  |
| 2 | …Scientology besitzt großen Einfluss in der BRD; verschiedene Großunternehmen gehören zu Scientology. |  |  |  |  |  |  |
| 3 | …in der ehemaligen UDSSR gab es mehrere schwere vertuschte Atomkraftunfälle. |  |  |  |  |  |  |
| 4 | … die wahre Geschichte hinter den Anschlägen vom 11. September 2001 entspricht nicht der von der Bush - Regierung verbreiteten Version. |  |  |  |  |  |  |
| 5 | … einflussreiche jüdische Familien kontrollieren große Bereiche des Weltgeschehens. |  |  |  |  |  |  |
| 6 | … Lady Di (Diana von Wales) wurde ermordet. |  |  |  |  |  |  |
| 7 | … die USA sind wegen des Öls im Jahr 2003 in den Irak einmarschiert. |  |  |  |  |  |  |
| 8 | … seit längerer Zeit haben verschiedene Regierungen Kontakt zu Außerirdischen. |  |  |  |  |  |  |

| 9 | … es gibt einen Geheimbund der "Illuminaten", deren Symbole das Allsehende Auge, die Pyramide und die Zahl "23" sind. |  |  |  |  |  |  |
| --- | --- | --- | --- | --- | --- | --- | --- |
| 10 | … Flugzeug-Kondensstreifen sind ab und an in Wirklichkeit Geheimversuche, sogenannte "Chemtrails", die die Umwelt schädigen. |  |  |  |  |  |  |
| 11 | … Jesus hat mit Maria Magdalena Kinder gezeugt, was von der Kirche vertuscht wird. |  |  |  |  |  |  |
| 12 | … das World Trade Center stürzte vor allem ein, weil es von innen gesprengt wurde |  |  |  |  |  |  |
| 13 | … es gibt verschiedene religiöse Gruppen, die Menschenopfer durchführen. |  |  |  |  |  |  |
| 14 | … die Automobilindustrie verzichtet auf den Einsatz von rostfreiem Stahl bei Auspuffanlagen nur deshalb, weil das die Umsätze mit deren regelmäßigem Austausch gefährden würde. |  |  |  |  |  |  |
| 15 | …es gibt religiöse Sekten, die die vollständige Kontrolle über die Psyche ihrer Mitglieder haben. |  |  |  |  |  |  |
| 16 | …hinter verschiedenen Geschehnissen der Weltgeschichte stehen in Wirklichkeit die Freimaurer. |  |  |  |  |  |  |
| 17 | … die Pharmaindustrie blockiert die Verbreitung gewisser sinnvoller Medikamente. |  |  |  |  |  |  |
| 18 | … die Nazis haben im Zweiten Weltkrieg funktionierende Flugscheiben in UFO-Optik entwickelt. |  |  |  |  |  |  |
| 19 | … in den USA gab es mehrere schwere vertuschte Atomkraftunfälle. |  |  |  |  |  |  |
| 20 | … eine kleine Gruppe von Personen lenkt die Geschicke der Erde. |  |  |  |  |  |  |

**Table S3: English translation of the conspiracy beliefs**

Please indicate whether you endorse the following conspiracy theories. The use of the term conspiracy theory does not indicate whether the theory is correct. This classification means that the theory contrasts with official representations of events.

I think...

1. J. F. Kennedy was not shot by Lee Harvey Oswald (alone).
2. Scientology has great influence in the federal republic of Germany; various large companies belong to Scientology.
3. In the former Union of Soviet Socialists Republic (USSR), there were several serious covered-up nuclear power accidents.
4. The true story behind the attacks of 11 September 2001 does not correspond to the version disseminated by the Bush government.
5. influential Jewish families control large parts of world affairs.
6. Lady Di (Diana of Wales) was murdered.
7. The USA invaded Iraq in 2003 to gain access to oil.
8. For some time, various governments have had contact with aliens.
9. There is a secret society of "Illuminati", whose symbols are the all-seeing eye, the pyramid and the number "23".
10. In reality, airplane condensation trails, so-called "chemtrails", which damage the environment, are secret experiments.
11. Jesus and Mary Magdalene fathered children, which is being covered up by the church.
12. The World Trade Center collapsed mainly because it was blown up from the inside.
13. There are various religious groups that perform human sacrifices.
14. The automotive industry is only abandoning the use of stainless steel in exhaust systems because its regular replacement jeopardizes sales.
15. There are religious sects that have complete control over the psyche of their members.
16. The various events that have occurred throughout world history are actually freemasons.
17. The pharmaceutical industry blocks the distribution of certain useful drugs.
18. Nazis developed functioning UFO-optic flying discs during World War II.
19. In the US, several serious nuclear accidents have occurred.
20. A small group of people directs the fate of the earth.

**Table S4: Statistical analyses**

Following the central limit theorem, variables in samples larger than *n* = 30 can be deemed normally distributed (Barri, 2019; Weinberg & Abramowitz, 2016). All analyses were computed with SPSS version 29. First, Pearson correlation coefficients were used to assess whether endorsement of conspiracy beliefs (*Conspiracy Beliefs Scale* (CB)), populist attitudes (*Generalist Populist Attitudes Scale* (Gen), *Anti-Elitist Attitudes Scale* (Anti), preference of unrestricted popular sovereignty (*Preference of Unrestricted Popular Sovereignty Scale* (Sov), belief in the homogeneity and virtuousness of the people (*Belief in the Homogeneity and Virtuousness of the People Scale* (Hom)) and the two thinking preferences scales, the scale assessing the preference for analytical thinking (*Need for Cognition Scale* (NC)) and the preference for intuitive thinking (*Faith in Intuition Scale* (FI)) were associated with age and education level. Group differences in sex assigned at birth (male, female, diverse) regarding CB, Gen, Anti, Hom, Sov, NC, and FI were assessed using univariate ANOVAs with Tukey’s honestly significant difference (HSD) *post hoc* tests.

Regarding explorative research question 1, we examined the association between endorsement of conspiracy beliefs (CB) and generalist populist attitudes (Gen) using Pearson's correlation coefficients, controlling for the covariates age, education level, and sex assigned at birth. The analysis was repeated without controlling for covariates, including persons with diverse sex assigned at birth (*n* = 5), and using age, education level, and sex assigned at birth as two dummy-coded covariates (male: yes/no and female: yes/no).

The explorative research question 2 was assessed using a hierarchical linear regression analysis (Enter method). Endorsement of conspiracy beliefs (CB) was included as a criterion variable, and the three populist attitudes (Anti, Sov & Hom) and putative covariates (age, sex assigned at birth, and education level) were included as predictors. In addition, we repeated the linear regression analysis without controlling for covariates and controlling for covariates, including persons with diverse sex assigned at birth (*n* = 5) and using age, education level, and sex assigned at birth as two dummy-coded covariates (male: yes/no and female: yes/no).

The explorative research question 3 was first assessed using hierarchical linear regression analyses (Enter method). All predictors were mean-centered. Endorsement of conspiracy beliefs (CB) was used as the criterion variable, and a preference for an analytical thinking style (Need for Cognition (NC)) and one of the three populist subscales (Anti, Hom or Sov) as predictors in the first block and the interaction term (interaction between a preference of an analytical thinking style (NC) and populist attitudes subscales (Anti, Hom or Sov) was included in the second step of the hierarchical linear regression analysis. Additional hierarchical linear regression analyses were performed using the endorsement of conspiracy beliefs (CB) as a criterion and including a preference for intuitive thinking style (Faith in Intuition (FI)) and one of the three populist subscales (Anti, Hom or Sov) as predictors in the first block and the interaction term (interaction between a preference on an intuitive thinking style (FI) and populist attitudes (Anti, Hom or Sov) in the second step of the hierarchical regression analysis. All hierarchical linear analyses were performed in two ways: 1) using age, education level, and sex assigned at birth as covariates, 2) without controlling for covariates, and 3) including persons with diverse sex assigned at birth (*n* = 5) and using age, education level and sex assigned at birth as two dummy-coded covariates.

Significant moderation effects were analyzed using the Johnson‒Neyman technique (Johnson & Fay, 1950) included in the PROCESS macro for SPSS (model 1) (Hayes & Preacher, 2014), a follow-up method for regressions containing interaction coefficients that identify over what moderator range a predictor has significant versus nonsignificant effects on the outcome measure (Bauer & Curran, 2005). Additionally, to ensure meaningful interpretations of the data, we used non-centered scores to generate and inspect a visual graph of the interaction effects. The required assumptions of both regression analyses (independence of errors and absence of multicollinearity) were tested by calculating the Durbin–Watson statistic and the variance inflation factors.

**Table S5: Sociodemographic characteristics (*n* = 483 participants)**

| *Sociodemographics* | *M/*(%)/*n* | *SD* |
| --- | --- | --- |
| Sex assigned at birth  female  male | 285 (58.40 %)  198 (40.57 %) |  |
| Age (in years) | 28.08 | 7.82 |
| Education level |  |  |
| No graduation | 1 (0.2 %) |  |
| Other degree ^1^ | 2 (0.4 %) |  |
| 9 school years ^2^ | 13 (2.7 %) |  |
| 10 school years ^3^ | 38 (7.9 %) |  |
| High school equivalent | 134 (27.7 %) |  |
| University degree (not specified) | 289 (59.8 %) |  |
| Ph.D. | 6 (1.2 %) |  |
| Affiliation to religious group |  |  |
| Roman-catholic | 174 (36.0 %) |  |
| Protestant | 128 (26.5 %) |  |
| Jewish | 3 (0.6 %) |  |
| Muslim | 10 (2.1 %) |  |
| Buddhist | 1 (0.2 %) |  |
| Jehovah’s witness | 1 (0.2 %) |  |
| Other Christian community | 7 (1.4 %) |  |
| Other | 5 (1.0 %) |  |
| No religion | 154 (31.9 %) |  |
| Religiosity | 1.83 | 0.84 |
| Political party to elect ^4^ |  |  |
| CDU/CSU ^5^ | 54 (11.2 %) |  |
| SPD ^6^ | 28 (5.8 %) |  |
| Green party | 198 (41.0 %) |  |
| FDP ^7^ | 58 (12.0 %) |  |
| AfD ^8^ | 7 (1.4 %) |  |
| Die Linke ^9^ | 57 (11.8 %) |  |
| Other | 40 (8.3 %) |  |
| I would not attend the election | 41 (8.5 %) |  |
| Diagnosis of mental disorder in the past ^10^ | Yes: 88 (18.22 %) |  |
| Depression | 59 (12.22 %) |  |
| Anxiety disorder | 25 (5.18 %) |  |
| Posttraumatic Stress Disorder | 10 (2.07 %) |  |
| Somatoform Disorder | 2 (0.41 %) |  |
| Eating disorder | 9 (1.86 %) |  |
| Obsessive-Compulsive disorder | 6 (1.24 %) |  |
|  |  |  |
| Endorsement of Conspiracy Beliefs Mean Score Scale (CB)  (*n* = 484) | 2.63 | 0.76 |
| Generalist Populist Attitudes mean score Scale (Gen) | 2.94 | 0.55 |
| Anti-Elitist Attitudes Scale (Anti) ^12^ | 3.31 | 0.73 |
| …Preference of Unrestricted Popular Sovereignty  Scale (Sov) | 3.25 | 0.88 |
| Belief in the Homogeneity and Virtuousness of the   People Scale (Hom) | 2.42 | 0.66 |
| Need for Cognition Scale (NC) | 5.04 | 0.94 |
| Faith in Intuition Scale (FI) | 4.10 | 0.81 |
|  |  |  |

Notes: ^1^ = another degree, e.g., special education degree; ^2^ = German Hauptschulabschluss (9 school years), ^3^ = German Realschulabschluss (10 school years), ^4^ = “If the election of the Bundestag would take place on Sunday, which party would you elect?”; ^5^ = Christliche Demokratische Union/Christliche Soziale Union (conservatives); ^6^ = Sozialdemokratische Partei Deutschland = middle-leftist party; ^7^ = Freie Demokratische Partei = conservative and liberal; ^8^ = Alternative für Deutschland = right-wing extremist, critical toward migration; ^9^ Die Linke = left-wing extremist party, former SED (Soziale Einheitspartei Deutschlands) from Eastern Germany; ^10^ = it was possible to choose several options.

**Table S6: Group differences in groups regarding sex assigned at birth in endorsement of conspiracy beliefs, populist attitudes, and analytical and intuitive thinking style**

|  | *Total group* | | *Male* | | *Female* | | *Group differences* |
| --- | --- | --- | --- | --- | --- | --- | --- |
|  | *n* | *M (SD)* | *n* | *M (SD)* | *n* | *M (SD)* | *Univariate ANOVA* |
| Endorsement of Conspiracy Beliefs (CB) | 479 | 2.63 (0.76) | 197 | 2.57 (0.83) | 282 | 2.68 (0.71) | *F (*1,477) = 2.563, *p* = .110 |
| Generalist Populist Attitudes Scale (Gen) | 483 | 2.94 (0.55) | 198 | 2.88 (0.58) | 285 | 2.99 (0.53) | *F (*1,481) = 4.434, *p* = .036 |
| Anti-Elitist attitudes Scale (Anti) | 483 | 3.32 (0.73) | 198 | 3.32 (0.78) | 285 | 3.31 (0.69) | *F (*1,481) = .014, *p* = .907 |
| Preference of Unrestricted | 483 | 3.25 (0.88) | 198 | 3.13 (0.98) | 285 | 3.33 (0.79) | *F (*1,481) = 6.155, *p* = .013 |
| Popular Sovereignty Scale (Sov) |  |  |  |  |  |  |  |
| Belief in the Homogeneity and Virtuousness of the People Scale (Hom) | 483 | 2.43 (0.66) | 198 | 2.34 (0.63) | 285 | 2.48 (0.68) | *F (*1,481) = 5.401, *p* = .021 |
| Need for Cognition Scale (NC) | 483 | 5.05 (0.95) | 198 | 5.25 (1.01) | 285 | 4.91 (0.87) | *F* (1,481) = 15.770, *p* < .001 |
| Faith in Intuition Scale (FI) | 483 | 4.10 (0.81) | 198 | 3.96 (0.87) | 285 | 4.19 (0.76) | *F (*1,481) = 10.248, *p* = .001 |

Notes: M = Mean; SD = Standard deviation.

**Table S7: Correlations between age, education level, and endorsement of conspiracy beliefs, populist attitudes, and analytical and intuitive thinking styles**

|  | Age (in years) | Education level |
| --- | --- | --- |
| Endorsement of Conspiracy Belief Scale (CB) | .110; *p* = .016 ^1^ | -.159; *p* < .001 ^1^ |
| Generalist Populist Attitudes Scale (Gen) | .037; *p* = .412 ^2^ | -.218; *p* < .001 ^2^ |
| Anti-Elitist Attitudes Scale (Anti) | .072; *p* = .116 ^2^ | -.178; *p* < .001 ^2^ |
| Preference of Unrestricted Popular | .022; *p* = .630 ^2^ | -.148; *p* = .001 ^2^ |
| Sovereignty Scale (Sov) |  |  |
| Belief in the Homogeneity and Virtuousness of the People Scale  (Hom) | -.007; *p* = .879 ^2^ | -.161; *p* < .001 ^2^ |
| Need for Cognition Scale (NC) | .121; *p* = .008 ^2^ | .076; *p* = .094 ^2^ |
| Faith in Intuition Scale (FI) | -.103; *p* = .023 ^2^ | -.096; *p* = .034 ^2^ |

Notes: M = Mean; SD = Standard deviation; ^1^ *n* = 479; ^2^ *n* = 483.

**Table S8: Correlations between endorsement of conspiracy beliefs, populist attitudes, and analytical and intuitive thinking styles controlling for age, education level, and sex assigned at birth, including persons with diverse sex assigned at birth**

|  |  | CB | Gen | Anti | Sov | Hom | FI | NC |
| --- | --- | --- | --- | --- | --- | --- | --- | --- |
| 1 | Conspiracy Belief Scale (CB) | 1 |  |  |  |  |  |  |
| 2 | Generalist Populist Attitudes Scale (Gen) | .403**^1^ | 1 |  |  |  |  |  |
| 3 | Anti-elitist Attitudes Scale (Anti) | .370**^1^ | .759**^2^ | 1 | . |  | . |  |
| 4 | Preference for Unrestricted Popular | .304**^1^ | 788**^2^ | 533**^2^ | 1 |  |  |  |
|  | Sovereignty (Sov) |  |  |  |  |  |  |  |
| 5 | Belief in the Homogeneity and Virtuousness of the People Scale (Hom) | .222**^1^ | .666**^2^ | .171**^2^ | .248**^2^ | 1 |  |  |
| 6 | Faith in Intuition (FI) | .360**^1^ | .347**^2^ | .267**^2^ | .246**^2^ | .254**^2^ | 1 |  |
| 7 | Need for Cognition (NC) | -.181**^1^ | -.346**^2^ | -.240**^2^ | -.196**^2^ | -.321**^2^ | -.327**^2^ | 1 |

Notes: ^**^: p < 0.001; ^1^ *n* = 484; ^2^ *n* = 488; CB = Endorsement of Conspiracy Beliefs Scale; Gen = Generalist Populist Attitudes Scale; Anti = Anti-elitist Attitudes Scale; Sov = Preference for Unrestricted Popular Sovereignty Scale; Hom = Belief in the Homogeneity and Virtuousness of the People Scale; NC = Need for Cognition Scale; FI = Faith in Intuition Scale.

**Table S9: Correlations between endorsement of conspiracy beliefs, populist attitudes, and analytical and intuitive thinking styles without controlling for covariates**

|  | CB | Gen | Anti | Sov | Hom | NC | FI |
| --- | --- | --- | --- | --- | --- | --- | --- |
| Endorsement of Conspiracy Belief Scale (CB) | 1 |  |  |  |  |  |  |
| Generalist Populist Attitudes Scale (Gen) | .430**^1^ | 1 |  |  |  |  |  |
| Anti-Elitist Attitudes Scale (Anti) | .393**^1^ | .765**^2^ | 1 |  |  |  |  |
| Preference for Unrestricted Popular | .332**^1^ | .796**^2^ | .545**^2^ | 1 |  |  |  |
| Sovereignty Scale (Sov) |  |  |  |  |  |  |  |
| Belief in the Homogeneity and virtuousness of the people Scale (Hom) | .244**^1^ | .684**^2^ | .199**^2^ | .279**^2^ | 1 |  |  |
| Need for Cognition Scale (NC) | -.181**^1^ | -.362**^2^ | -.238**^2^ | -.222**^2^ | -.342**^2^ | 1 |  |
| Faith in Intuition Scale (FI) | .361**^1^ | .366**^2^ | .275**^2^ | .269**^2^ | .275**^2^ | -.363**^2^ | 1 |

Notes: ^**^: p < 0.001; ^1^ *n* = 479; ^2^ *n* = 483; CB = Endorsement of conspiracy beliefs Scale; Gen = Generalist Populist attitudes Scale; Anti = Anti-elitist Attitudes Scale; Sov = Preference for unrestricted popular sovereignty Scale; Hom = Belief in the Homogeneity and virtuousness of the people Scale; NC = Need for Cognition Scale; FI = Faith in intuition Scale.

**Table S10: Linear regression analysis predicting endorsement of conspiracy belief controlling for age, education level, and sex assigned at birth, including persons with diverse sex**

|  |  | *Unstandardized  coefficients* | | *Standardized coefficients* |  | |  | |  | | |  | | |
| --- | --- | --- | --- | --- | --- | --- | --- | --- | --- | --- | --- | --- | --- | --- |
| Step | Predictor | *B* | *SE* | *Beta* | | *p* | | *R^2^* | | *R^2 change^* | *F/F ^change^ (df1/df2)* | | *p* |  |
| 1 |  |  |  |  | |  | | .048 | | .048 | *F* (4,479) = 6.005 | | < .001 |  |
|  | Age | .011 | .004 | .114 | | .012 | |  | |  |  | |  |  |
|  | Male ^1^ | -.529 | .340 | -.340 | | .120 | |  | |  |  | |  |  |
|  | Female ^1^ | -.393 | .339 | -.253 | | .247 | |  | |  |  | |  |  |
|  | Education level | -.143 | .041 | -.155 | | < .001 | |  | |  |  | |  |  |
| 2 |  |  |  |  | |  | | .212 | | .164 | *F* (3,476) = 33.078 | | < .001 |  |
|  | Age | .009 | .004 | .093 | | .024 | |  | |  |  | |  |  |
|  | Male ^1^ | -.659 | .311 | -.423 | | .035 | |  | |  |  | |  |  |
|  | Female ^1^ | -.570 | .310 | -.367 | | .066 | |  | |  |  | |  |  |
|  | Education level | -.057 | .039 | -.062 | | .138 | |  | |  |  | |  |  |
|  | Anti | .295 | .051 | .282 | | < .001 | |  | |  |  | |  |  |
|  | Sov | .102 | .043 | .117 | | .020 | |  | |  |  | |  |  |
|  | Hom | .166 | .050 | .143 | | < .001 | |  | |  |  | |  |  |

Notes: SE = standardized error of B; Anti = Anti-elitist Attitudes scale; Sov = Preference for Unrestricted Popular Sovereignty Scale; Hom = Belief in the Homogeneity and Virtuousness of the People Scale; ^1^ = Sex assigned at birth has three options (male, female, diverse): the variable was dummy-coded

**Table S11: Linear regression analysis on populistic attitudes predicting endorsement of conspiracy beliefs without controlling for covariates**

|  | *Unstandardised coefficients* | |  | *Standardised coefficients* | |  |  |  |
| --- | --- | --- | --- | --- | --- | --- | --- | --- |
| Predictor | *B* | *SE* |  | *Beta* | *p* | *R^2^* | *F (df1, df2)* | *p* |
|  |  |  |  |  |  | .190 | *F* (3,474) = 38.304 | < .001 |
| Anti | .305 | .051 |  | .292 | < .001 |  |  |  |
| Sov | .114 | .044 |  | .131 | .009 |  |  |  |
| Hom | .172 | .050 |  | .149 | < .001 |  |  |  |

Note: SE = standardized error of B; Anti = Anti-elitist Attitudes Scale; Sov = Preference for Unrestricted Popular Sovereignty Scale; Hom = Belief in the Homogeneity and Virtuousness of the People Scale.

**Table S12: Moderator analysis (hierarchical linear regression analysis) assessing whether anti-elitist attitudes moderate the association between a preference for an analytical thinking style and endorsement of conspiracy beliefs, including persons with diverse sex**

|  |  | *Unstandardized coefficients* | | *Standardized coefficients* | |  |  |  |  |
| --- | --- | --- | --- | --- | --- | --- | --- | --- | --- |
| Step | Predictor | *B* | *SE* | *Beta* | *p* | *R^2^* | *R^2 change^* | *F/F ^change^ (df1/df2)* | *p* |
| 1 |  |  |  |  |  | .187 | .187 | *F* (6, 477) = 18.289 | < .001 |
|  | Age | .010 | .004 | .103 | .014 |  |  |  |  |
|  | Male^1^ | -.611 | .316 | -.392 | .054 |  |  |  |  |
|  | Female^1^ | -.504 | .315 | -.325 | .110 |  |  |  |  |
|  | Education level | -.078 | .039 | -.084 | .046 |  |  |  |  |
|  | NC | -.080 | .036 | -.098 | .025 |  |  |  |  |
|  | Anti | .361 | .045 | .346 | < .001 |  |  |  |  |
| 2 |  |  |  |  |  | .205 | .018 | *F* (1, 476) = 10.878 | .001 |
|  | Age | .010 | .004 | .101 | .016 |  |  |  |  |
|  | Male^1^ | -.558 | .314 | -.358 | .076 |  |  |  |  |
|  | Female^1^ | -.466 | .312 | -.300 | .136 |  |  |  |  |
|  | Education level | -.068 | .039 | -.074 | .077 |  |  |  |  |
|  | NC | -.099 | .036 | -.121 | .006 |  |  |  |  |
|  | Anti | .199 | .066 | .191 | .003 |  |  |  |  |
|  | Interaction Anti x NC | .146 | .044 | .204 | .001 |  |  |  |  |

Notes: SE = standardized error of B; NC = Need for Cognition scale; Anti = Anti-elitist Attitudes Scale; ^1^ = sex assigned at birth has three options (male, female, diverse): the variable was dummy-coded

**Table S13: Moderator analysis (hierarchical linear regression analysis) assessing whether anti-elitist attitudes moderate the association between a**

**preference for an analytical thinking style and endorsement of conspiracy beliefs without controlling for covariates**

|  |  | *Unstandardised coefficients* | | *Standardised coefficients* | |  |  |  |  |
| --- | --- | --- | --- | --- | --- | --- | --- | --- | --- |
| Step | Predictor | *B* | *SE* | *Beta* | *p* | *R^2^* | *R^2 change^* | *F (df1,df2)/F ^change^* | *p* |
| 1 |  |  |  |  |  | .163 | .163 | *F* (2,476) = 46.298 | < .001 |
|  | NC | -.076 | .035 | -.093 | .032 |  |  |  |  |
|  | Anti | .388 | .045 | .371 | <.001 |  |  |  |  |
| 2 |  |  |  |  |  | .183 | .020 | *F* (1,475) = 11.547 | < .001 |
|  | NC | -.095 | .035 | -.117 | .007 |  |  |  |  |
|  | Anti | .217 | 067 | .208 | .001 |  |  |  |  |
|  | Interaction NC x Anti | .153 | .045 | .213 | < .001 |  |  |  |  |

Note: SE = standardized error of B; Anti = Anti-elitist Attitudes Scale; NC = Need for Cognition Scale.

**Table S14: Moderator analysis (hierarchical linear regression analysis) assessing whether the belief in the homogeneity and virtuousness of the people moderates the association between a preference for an analytical thinking style and endorsement of conspiracy beliefs, controlling for age, education level, and sex assigned at birth**

|  |  | *Unstandardized coefficients* | |  | *Standardized coefficients* | |  |  |  |  |
| --- | --- | --- | --- | --- | --- | --- | --- | --- | --- | --- |
| Step | Predictor | *B* | *SE* |  | *Beta* | *p* | *R^2^* | *R^2 change^* | *F/F ^change^ (df1/df2)* | *p* |
| 1 |  |  |  |  |  |  | .101 | .101 | *F* (5, 473) = 10.585 | < .001 |
|  | Age | .012 | .004 |  | .126 | .005 |  |  |  |  |
|  | Sex assigned at birth | .074 | .069 |  | .048 | .287 |  |  |  |  |
|  | Education level | -.105 | .041 |  | -.113 | .011 |  |  |  |  |
|  | NC | -.096 | .036 |  | -.118 | .013 |  |  |  |  |
|  | Hom | .209 | .046 |  | .181 | < .001 |  |  |  |  |
| 2 |  |  |  |  |  |  | .093 | .003 | *F* (1, 472) = 1.791 | .181 |
|  | Age | .013 | .004 |  | .130 | .004 |  |  |  |  |
|  | Sex assigned at birth | .076 | .069 |  | .049 | .271 |  |  |  |  |
|  | Education level | -.105 | .041 |  | -.114 | .010 |  |  |  |  |
|  | NC | -.100 | .039 |  | -.123 | .010 |  |  |  |  |
|  | Hom | .139 | .075 |  | .120 | .065 |  |  |  |  |
|  | Interaction NC x Hom | .069 | .052 |  | .083 | .181 |  |  |  |  |

Notes: SE = standardized error of B; NC = Need for Cognition Scale; Hom = Belief in the Homogeneity and Virtuousness of the People Scale;

**Table S15: Moderator analysis (hierarchical linear regression analysis) assessing whether beliefs in the homogeneity and virtuousness of the people moderate the association between a preference for an analytical thinking style and endorsement of conspiracy beliefs, controlling for age, education level, and sex assigned at birth, including persons with diverse sex assigned at birth**

|  |  | *Unstandardized coefficients* | |  | *Standardized coefficients* | |  |  |  |  |
| --- | --- | --- | --- | --- | --- | --- | --- | --- | --- | --- |
| Step | Predictor | *B* | *SE* |  | *Beta* | *p* | *R^2^* | *R^2 change^* | *F/F ^change^ (df1/df2)* | *p* |
| 1 |  |  |  |  |  |  | .107 | .107 | *F* (6, 477) = 9.552 | < .001 |
|  | Age | .012 | .004 |  | .127 | .004 |  |  |  |  |
|  | Male^1^ | -.484 | .331 |  | -.311 | .144 |  |  |  |  |
|  | Female^1^ | -.412 | .330 |  | -.266 | .212 |  |  |  |  |
|  | Education level | -.105 | .041 |  | -.114 | .010 |  |  |  |  |
|  | NC | -.100 | .038 |  | -.123 | .009 |  |  |  |  |
|  | Hom | .211 | .054 |  | .182 | < .001 |  |  |  |  |
| 2 |  |  |  |  |  |  | .110 | .003 | *F* (1, 476) = 1.664 | .198 |
|  | Age | .013 | .004 |  | .130 | .003 |  |  |  |  |
|  | Male^1^ | -.485 | .331 |  | -.312 | .143 |  |  |  |  |
|  | Female^1^ | -.411 | .329 |  | -.265 | .213 |  |  |  |  |
|  | Education level | -.105 | .041 |  | -.114 | .010 |  |  |  |  |
|  | NC | -.104 | .038 |  | -.128 | .007 |  |  |  |  |
|  | Hom | .144 | .075 |  | .124 | .055 |  |  |  |  |
|  | Interaction NC x Hom | .066 | .051 |  | .079 | .198 |  |  |  |  |

Notes: SE = standardized error of B; NC = Need for Cognition Scale; Hom = Belief in the Homogeneity and Virtuousness of the People Scale; ^1^ = Sex assigned at birth has three options (male, female, diverse): the variable was dummy-coded

**Table S16: Moderator analysis (hierarchical linear regression analysis) assessing whether the belief in the homogeneity and virtuousness of the people moderates the association between a preference for an analytical thinking style and endorsement of conspiracy beliefs, without controlling for covariates**

|  |  | *Unstandardised coefficients* | | *Standardised coefficients* | |  |  |  |  |
| --- | --- | --- | --- | --- | --- | --- | --- | --- | --- |
| Step | Predictor | *B* | *SE* | *Beta* | *p* | *R^2^* | *R^2 change^* | *F (df1,df2)/F ^change^* | *p* |
| 1 |  |  |  |  |  | .070 | .070 | *F* (2,476) = 18.012 | < .001 |
|  | NC | -.091 | .038 | -.112 | .018 |  |  |  |  |
|  | Hom | .238 | .054 | .204 | < .001 |  |  |  |  |
| 2 |  |  |  |  |  | .073 | .002 | *F* (1,475) = 1.250 | .264 |
|  | NC | -.095 | .038 | -.116 | .014 |  |  |  |  |
|  | Hom | .180 | .075 | .155 | .018 |  |  |  |  |
|  | Interaction Hom x NC | .058 | .052 | .070 | .264 |  |  |  |  |

Note: SE = standardized error of B; NC = Need for Cognition Scale; Hom = Belief in the Homogeneity and Virtuousness of the People Scale.

**Table S17: Moderator analysis (hierarchical linear regression analysis) assessing whether a preference for unrestricted popular sovereignty moderates the association between a preference for an analytical thinking style and endorsement of conspiracy beliefs, controlling for age, education level, and sex assigned at birth and including persons with diverse sex assigned at birth**

|  |  | *Unstandardized coefficients* | |  | *Standardized coefficients* | |  |  |  |  |
| --- | --- | --- | --- | --- | --- | --- | --- | --- | --- | --- |
| Step | Predictor | *B* | *SE* |  | *Beta* | *p* | *R^2^* | *R^2 change^* | *F/F ^change^ (df1/df2)* | *p* |
| 1 |  |  |  |  |  |  | .150 | .150 | *F* (6, 477) = 14.066 | < .001 |
|  | Age | .012 | .004 |  | .120 | .006 |  |  |  |  |
|  | Male^1^ | -.457 | .323 |  | -.294 | .157 |  |  |  |  |
|  | Female^1^ | -.408 | .321 |  | -.263 | .205 |  |  |  |  |
|  | Education level | -.093 | .040 |  | -.101 | .019 |  |  |  |  |
|  | NC | -.103 | .036 |  | -.127 | .004 |  |  |  |  |
|  | Sov | .242 | .038 |  | .278 | < .001 |  |  |  |  |
| 2 |  |  |  |  |  |  | .160 | .010 | *F* (1, 476) = 5.488 | .020 |
|  | Age | .011 | .004 |  | .117 | .006 |  |  |  |  |
|  | Male^1^ | -.425 | .321 |  | -.273 | .187 |  |  |  |  |
|  | Female^1^ | -.381 | .320 |  | -.246 | .234 |  |  |  |  |
|  | Education level | -.083 | .040 |  | -.090 | .036 |  |  |  |  |
|  | NC | -.119 | .037 |  | -.146 | .001 |  |  |  |  |
|  | Sov | .137 | .059 |  | .158 | .020 |  |  |  |  |
|  | Interaction NC x Sov | .089 | .038 |  | .155 | .020 |  |  |  |  |

Notes: SE = standardized error of B; NC = Need for Cognition Scale; Sov = Preference for Unrestricted Popular Sovereignty Scale; ^1^ = Sex assigned at birth has three options (male, female, diverse): the variable was dummy-coded

**Table S18: Moderator analysis (hierarchical linear regression analysis) assessing whether the preference for unrestricted popular sovereignty moderates the association between a preference for an analytical thinking style and endorsement of conspiracy beliefs without controlling for covariates**

|  |  | *Unstandardised coefficients* | |  | *Standardised coefficients* | |  |  |  |  |
| --- | --- | --- | --- | --- | --- | --- | --- | --- | --- | --- |
| Step | Predictor | *B* | *SE* |  | *Beta* | *p* | *R^2^* | *R^2 change^* | *F (df1,df2)/F ^change^* | *p* |
| 1 |  |  |  |  |  |  | .122 | .122 | *F* (2,476) = 33.161 | < .001 |
|  | NC | -.092 | .036 |  | -.113 | .010 |  |  |  |  |
|  | Sov | .266 | .038 |  | .307 | < .001 |  |  |  |  |
| 2 |  |  |  |  |  |  | .134 | .012 | *F* (1,475) = 6.529 | .011 |
|  | NC | -.110 | .036 |  | -.135 | .003 |  |  |  |  |
|  | Sov | .137 | .059 |  | .171 | .013 |  |  |  |  |
|  | Interaction NC x Sov | .103 | .038 |  | .172 | .011 |  |  |  |  |

Note: SE = standardized error of B; NC = Need for Cognition Scale; Sov = Preference for Unrestricted Popular Sovereignty Scale.

**Table S19: Moderator analysis (hierarchical linear regression analysis) assessing whether anti-elitist attitudes moderate the association between a preference for an intuitive thinking style and endorsement of conspiracy beliefs, controlling for age, education level, and sex assigned at birth**

|  |  | *Unstandardized coefficients* | |  | *Standardized coefficients* | |  |  |  |  |
| --- | --- | --- | --- | --- | --- | --- | --- | --- | --- | --- |
| Step | Predictor | *B* | *SE* |  | *Beta* | *p* | *R^2^* | *R^2 change^* | *F/F ^change^ (df1/df2)* | *p* |
| 1 |  |  |  |  |  |  | .184 | .184 | *F* (5, 473) = 21.300 | < .001 |
|  | Age | .014 | .004 |  | .144 | < .001 |  |  |  |  |
|  | Sex assigned at birth | .042 | .066 |  | .027 | .526 |  |  |  |  |
|  | Education level | -.090 | .039 |  | -.097 | .022 |  |  |  |  |
|  | FI | .306 | .041 |  | .325 | < .001 |  |  |  |  |
|  | Anti | .158 | .051 |  | .137 | .002 |  |  |  |  |
| 2 |  |  |  |  |  |  | .186 | .002 | *F* (1, 472) = 1.214 | .271 |
|  | Age | .014 | .004 |  | .148 | < .001 |  |  |  |  |
|  | Sex assigned at birth | .041 | .066 |  | .026 | .534 |  |  |  |  |
|  | Education level | -.091 | .039 |  | -.099 | .020 |  |  |  |  |
|  | FI | .300 | .042 |  | .318 | < .001 |  |  |  |  |
|  | Anti | .163 | .051 |  | .140 | .001 |  |  |  |  |
|  | Interaction FI x Anti | -.062 | .057 |  | -.047 | .271 |  |  |  |  |

Notes: SE = standardized error of B; FI = Faith in Intuition Scale; Anti = Anti-elitist Attitudes Scale;

**Table S20: Moderator analysis (hierarchical linear regression analysis) assessing whether anti-elitist attitudes moderate the association between a**

**preference for an intuitive thinking style and endorsement of conspiracy beliefs, controlling for age, education level, and sex assigned at birth and including persons with diverse sex assigned at birth**

|  |  | *Unstandardized coefficients* | |  | *Standardized coefficients* | |  |  |  |  |
| --- | --- | --- | --- | --- | --- | --- | --- | --- | --- | --- |
| Step | Predictor | *B* | *SE* |  | *Beta* | *p* | *R^2^* | *R^2 change^* | *F/F ^change^ (df1/df2)* | *p* |
| 1 |  |  |  |  |  |  | .249 | .249 | *F* (6, 477) = 26.299 | < .001 |
|  | Age | .012 | .004 |  | .122 | .003 |  |  |  |  |
|  | Male^1^ | -.550 | .303 |  | -.354 | .070 |  |  |  |  |
|  | Female^1^ | -.478 | .302 |  | -.309 | .114 |  |  |  |  |
|  | Education level | -.067 | .037 |  | -.072 | .074 |  |  |  |  |
|  | FI | .265 | .040 |  | .281 | < .001 |  |  |  |  |
|  | Anti | .307 | .044 |  | .294 | < .001 |  |  |  |  |
| 2 |  |  |  |  |  |  | .239 | .002 | *F* (1, 476) = 1.054 | .305 |
|  | Age | .012 | .004 |  | .123 | .002 |  |  |  |  |
|  | Male^1^ | -.534 | .304 |  | -.343 | .080 |  |  |  |  |
|  | Female^1^ | -.461 | .303 |  | -.297 | .128 |  |  |  |  |
|  | Education level | -.069 | .037 |  | -.074 | .067 |  |  |  |  |
|  | FI | .264 | .040 |  | .280 | < .001 |  |  |  |  |
|  | Anti | .304 | .044 |  | .291 | < .001 |  |  |  |  |
|  | Interaction FI x Anti | -.048 | .047 |  | -.041 | .305 |  |  |  |  |

Notes: SE = standardized error of B; Anti = Anti-elitist Attitudes Scale; FI = Faith in Intuition Scale; ^1^ = sex assigned at birth has three options (male, female, diverse): the variable was dummy-coded.

**Table S21: Moderator analysis (hierarchical linear regression analysis) assessing whether anti-elitist attitudes moderate the association between a preference for intuitive thinking and endorsement of conspiracy beliefs without controlling for covariates**

|  |  | *Unstandardised coefficients* | |  | *Standardised coefficients* | |  |  |  |  |
| --- | --- | --- | --- | --- | --- | --- | --- | --- | --- | --- |
| Step | Predictor | *B* | *SE* |  | *Beta* | *p* | *R^2^* | *R^2 change^* | *F (df1,df2)/F ^change^* | *p* |
| 1 |  |  |  |  |  |  | .221 | .224 | *F* (2,476) = 68.787 | < .001 |
|  | FI | .258 | .039 |  | .274 | < .001 |  |  |  |  |
|  | Anti | .333 | .044 |  | .319 | < .001 |  |  |  |  |
| 2 |  |  |  |  |  |  | .220 | .001 | *F* (1,475) = .599 | .439 |
|  | FI | .257 | .040 |  | .274 | < .001 |  |  |  |  |
|  | Anti | .330 | .044 |  | .316 | < .001 |  |  |  |  |
|  | Interaction FI x Anti | -.037 | .048 |  | -0.31 | .439 |  |  |  |  |

Note: SE = standardized error of B; FI = Faith in Intuition Scale; Anti = Anti-elitist attitudes Scale.

**Table S22: Moderator analysis (hierarchical linear regression analysis) on whether beliefs in the homogeneity and virtuousness of the people moderate the association between a preference for intuitive thinking and endorsement of conspiracy beliefs, controlling for age, education level, and sex assigned at birth**

|  |  | *Unstandardized coefficients* | |  | *Standardized coefficients* | |  |  |  |  |
| --- | --- | --- | --- | --- | --- | --- | --- | --- | --- | --- |
| Step | Predictor | *B* | *SE* |  | *Beta* | *p* | *R^2^* | *R^2 change^* | *F/F ^change^ (df1/df2)* | *p* |
| 1 |  |  |  |  |  |  | .101 | .101 | *F* (5, 473) = 10.585 | < .001 |
|  | Age | .012 | .004 |  | .126 | .005 |  |  |  |  |
|  | Sex assigned at birth | .074 | .069 |  | .048 | .287 |  |  |  |  |
|  | Education level | -.105 | .041 |  | -.113 | .011 |  |  |  |  |
|  | FI | -.096 | .036 |  | -.118 | .013 |  |  |  |  |
|  | Hom | .209 | .046 |  | .181 | < .001 |  |  |  |  |
| 2 |  |  |  |  |  |  | .093 | .003 | *F* (1, 472) = 1.791 | .181 |
|  | Age | .013 | .004 |  | .130 | .004 |  |  |  |  |
|  | Sex assigned at birth | .076 | .069 |  | .049 | .271 |  |  |  |  |
|  | Education level | -.105 | .041 |  | -.114 | .010 |  |  |  |  |
|  | FI | -.100 | .039 |  | -.123 | .010 |  |  |  |  |
|  | Hom | .139 | .075 |  | .120 | .065 |  |  |  |  |
|  | Interaction FI x Hom | .069 | .052 |  | .083 | .181 |  |  |  |  |

Notes: SE = standardized error of B; FI = Faith in Intuition Scale; Hom = Belief in the Homogeneity and Virtuousness of the People Scale.

**Table S23: Moderator analysis (hierarchical linear regression analysis) assessing whether beliefs in the homogeneity and virtuousness of the people moderate the association between a preference for intuitive thinking and endorsement of conspiracy beliefs, controlling for age, education level, and sex assigned at birth and including persons with diverse sex assigned at birth**

|  |  | *Unstandardized coefficients* | |  | *Standardized coefficients* | |  |  |  |  |
| --- | --- | --- | --- | --- | --- | --- | --- | --- | --- | --- |
| Step | Predictor | *B* | *SE* |  | *Beta* | *p* | *R^2^* | *R^2 change^* | *F/F ^change^ (df1/df2)* | *p* |
| 1 |  |  |  |  |  |  | .189 | .189 | *F* (6, 477) = 18.483 | < .001 |
|  | Age | .014 | .004 |  | .143 | < .001 |  |  |  |  |
|  | Male^1^ | -.438 | .315 |  | -.281 | .165 |  |  |  |  |
|  | Female^1^ | -.396 | .314 |  | -.256 | .207 |  |  |  |  |
|  | Education level | -.090 | .039 |  | -.098 | .021 |  |  |  |  |
|  | FI | .306 | .041 |  | .324 | < .001 |  |  |  |  |
|  | Hom | .161 | .051 |  | .139 | .002 |  |  |  |  |
| 2 |  |  |  |  |  |  | .190 | .002 | *F* (1, 476) = 1.076 | .300 |
|  | Age | .014 | .004 |  | .147 | < .001 |  |  |  |  |
|  | Male^1^ | -.443 | .315 |  | -.285 | .160 |  |  |  |  |
|  | Female^1^ | -.402 | .314 |  | -.259 | .200 |  |  |  |  |
|  | Education level | -.091 | .039 |  | -.099 | .019 |  |  |  |  |
|  | FI | .300 | .042 |  | .318 | < .001 |  |  |  |  |
|  | Hom | .165 | .051 |  | .142 | .001 |  |  |  |  |
|  | Interaction FI x Hom | -.059 | .057 |  | -.044 | .300 |  |  |  |  |

Notes: SE = standardized error of B; FI = Faith in Intuition Scale; Hom = Belief in the Homogeneity and Virtuousness of the People Scale; ^1^ = sex assigned at birth has 3 options (male, female, diverse): the variable was dummy-coded

**Table S24: Moderator analysis (hierarchical linear regression analysis) assessing whether beliefs in the homogeneity and virtuousness of the people moderate the association between a preference for an intuitive thinking style and endorsement of conspiracy beliefs without controlling for covariates**

|  |  | *Unstandardised coefficients* | |  | *Standardised coefficients* | |  |  |  |  |
| --- | --- | --- | --- | --- | --- | --- | --- | --- | --- | --- |
| Step | Predictor | *B* | *SE* |  | *Beta* | *p* | *R^2^* | *R^2 change^* | *F (df1,df2)/F ^change^* | *p* |
| 1 |  |  |  |  |  |  | .153 | .153 | *F* (2,476) = 42.979 | < .001 |
|  | FI | .299 | .041 |  | .318 | < .001 |  |  |  |  |
|  | Hom | .181 | .051 |  | .157 | < .001 |  |  |  |  |
| 2 |  |  |  |  |  |  | .154 | .001 | *F* (1,475) = .392 | .471 |
|  | FI | .295 | .042 |  | .313 | < .001 |  |  |  |  |
|  | Hom | .185 | .051 |  | .160 | < .001 |  |  |  |  |
|  | Interaction FI x Hom | -.041 | .057 |  | -.031 | .471 |  |  |  |  |

Note: SE = standardized error of B; FI = Faith in Intuition Scale; Hom = Belief in the Homogeneity and Virtuousness of the People Scale

**Table S25: Moderator analysis (hierarchical linear regression analysis) on whether a preference for unrestricted popular sovereignty moderates the association between a preference for intuitive thinking and endorsement of conspiracy beliefs, controlling for age, education level, and sex assigned at birth**

|  |  | *Unstandardized coefficients* | |  | *Standardized coefficients* | |  |  |  |  |
| --- | --- | --- | --- | --- | --- | --- | --- | --- | --- | --- |
| Step | Predictor | *B* | *SE* |  | *Beta* | *p* | *R^2^* | *R^2 change^* | *F/F ^change^ (df1/df2)* | *p* |
| 1 |  |  |  |  |  |  | .184 | .184 | *F* (5, 473) = 21.300 | < .001 |
|  | Age | .014 | .004 |  | .144 | < .001 |  |  |  |  |
|  | Sex assigned at birth | .042 | .066 |  | .027 | .526 |  |  |  |  |
|  | Education level | -.090 | .039 |  | -.097 | .022 |  |  |  |  |
|  | FI | .306 | .041 |  | .325 | < .001 |  |  |  |  |
|  | Sov | .158 | .051 |  | .137 | .002 |  |  |  |  |
| 2 |  |  |  |  |  |  | .186 | .002 | *F* (1, 472) = 1.214 | .271 |
|  | Age | .014 | .004 |  | .148 | < .001 |  |  |  |  |
|  | Sex assigned at birth | .041 | .066 |  | .026 | .534 |  |  |  |  |
|  | Education level | -.091 | .039 |  | -.099 | .020 |  |  |  |  |
|  | FI | .300 | .042 |  | .318 | < .001 |  |  |  |  |
|  | Sov | .163 | .051 |  | .140 | .001 |  |  |  |  |
|  | Interaction FI x Sov | -.062 | .057 |  | -.047 | .271 |  |  |  |  |

Notes: SE = standardized error of B; FI = Faith in Intuition Scale; Sov = Preference for Unrestricted Popular Sovereignty.

**Table S26: Moderator analysis (hierarchical linear regression analysis) on whether the preference for unrestricted popular sovereignty moderates the association between a preference for an intuitive thinking style and endorsement of conspiracy beliefs, controlling for age, education level, and sex assigned at birth and including persons with diverse sex assigned at birth**

|  |  | *Unstandardized coefficients* | |  | *Standardized coefficients* | |  |  |  |  |
| --- | --- | --- | --- | --- | --- | --- | --- | --- | --- | --- |
| Step | Predictor | *B* | *SE* |  | *Beta* | *p* | *R^2^* | *R^2 change^* | *F/F ^change^ (df1/df2)* | *p* |
| 1 |  |  |  |  |  |  | .218 | .218 | *F* (6, 477) = 22.223 | < .001 |
|  | Age | .013 | .004 |  | .135 | < .001 |  |  |  |  |
|  | Male^1^ | -.426 | .309 |  | -.274 | .169 |  |  |  |  |
|  | Female^1^ | -.399 | .308 |  | -.257 | .195 |  |  |  |  |
|  | Education level | -.081 | .038 |  | -.088 | .033 |  |  |  |  |
|  | FI | .286 | .040 |  | .303 | < .001 |  |  |  |  |
|  | Sov | .199 | .037 |  | .228 | < .001 |  |  |  |  |
| 2 |  |  |  |  |  |  | .218 | <.0001 | *F* (1, 476) <.0001 | .983 |
|  | Age | .013 | .004 |  | .135 | .001 |  |  |  |  |
|  | Male^1^ | -.426 | .309 |  | -.274 | .169 |  |  |  |  |
|  | Female^1^ | -.399 | .308 |  | -.257 | .196 |  |  |  |  |
|  | Education level | -.081 | .038 |  | -.088 | .033 |  |  |  |  |
|  | FI | .286 | .040 |  | .303 | < .001 |  |  |  |  |
|  | Sov | .199 | .037 |  | .228 | < .001 |  |  |  |  |
|  | Interaction FI x Sov | .001 | .038 |  | .001 | .983 |  |  |  |  |

Notes: SE = standardized error of B; FI = Faith in Intuition Scale; Sov = Preference for Unrestricted Popular Sovereignty Scale; ^1^ = Sex assigned at birth has three options (male, female, diverse): the variable was dummy-coded

**Table S27: Moderator analysis (hierarchical linear regression analysis) on whether the preference for unrestricted popular sovereignty moderates the association between a preference for an intuitive thinking style and endorsement of conspiracy beliefs, without controlling for covariates**

|  |  | *Unstandardised coefficients* | |  | *Standardised coefficients* | |  |  |  |  |
| --- | --- | --- | --- | --- | --- | --- | --- | --- | --- | --- |
| Step | Predictor | *B* | *SE* |  | *Beta* | *p* | *R^2^* | *R^2 change^* | *F (df1,df2)/F ^change^* | *p* |
| 1 |  |  |  |  |  |  | .187 | .190 | *F* (2,476) = 55.869 | < .001 |
|  | FI | .276 | .040 |  | .293 | < .001 |  |  |  |  |
|  | Sov | .220 | .037 |  | .254 | < .001 |  |  |  |  |
| 2 |  |  |  |  |  |  | .185 | < .001 | *F* (1,475) < .001 | .984 |
|  | FI | .276 | .040 |  | .293 | < .001 |  |  |  |  |
|  | Sov | .220 | .037 |  | .254 | < .001 |  |  |  |  |
|  | Interaction FI x Sov | - .001 | .039 |  | - .001 | .984 |  |  |  |  |

Note: SE = standardized error of B; FI = Faith in Intuition Scale; Sov = Preference for Unrestricted Popular Sovereignty Scale.

**Table S28: Means and standard deviations of conspiracy beliefs**

|  | Conspiracy beliefs ^1^ | *n* | M (SD) |
| --- | --- | --- | --- |
| 1 | JFK | 342 | 2.95 (1.23) ^2^ |
| 2 | Scientology | 377 | 2.25 (1.18) ^2^ |
| 3 | Nuclear accidents UDSSR | 304 | 2.89 (1.17) ^2^ |
| 4 | 11^th^ September | 453 | 2.83 (1.32) ^2^ |
| 5 | Jewish families | 422 | 2.07 (1.24) ^2^ |
| 6 | Lady Di | 435 | 2.62 (1.32) ^2^ |
| 7 | Iraq invasion | 409 | 3.83 (0.99) ^2^ |
| 8 | Alien contact | 449 | 1.46 (0.91) ^2^ |
| 9 | Illuminati | 416 | 2.29 (1.33) ^2^ |
| 10 | Chemtrails | 434 | 1.46 (0.97) ^2^ |
| 11 | Marie Magdalene | 348 | 2.62 (1.35) ^2^ |
| 12 | World Trade Center | 444 | 2.16 (1.35) ^2^ |
| 13 | Human sacrifices | 391 | 3.45 (1.16) ^2^ |
| 14 | Stainless steel | 357 | 3.23 (1.16) ^2^ |
| 15 | Mind control | 452 | 3.95 (1.14) ^2^ |
| 16 | Freemasons | 362 | 2.07 (1.14) ^2^ |
| 17 | Pharmaceutical industry | 466 | 3.58 (1.26) ^2^ |
| 18 | Nazi UFOs | 336 | 1.82 (1.17) ^2^ |
| 19 | Nuclear accidents USA | 327 | 2.27 (1.11) ^2^ |
| 20 | Fate of Earth | 430 | 2.40 (1.37) ^2^ |

Notes: ^1^ See Tables S2 and S3 for the specific conspiracy belief; ^2^ range of the item between 1 and 5.

**Table S29: Intercorrelations between individual conspiracy beliefs**

|  | Conspiracy beliefs ^1^ | 1 | 2 | 3 | 4 | 5 | 6 | 7 | 8 | 9 | 10 | 11 | 12 | 13 | 14 | 15 | 16 | 17 | 18 | 19 | 20 |
| --- | --- | --- | --- | --- | --- | --- | --- | --- | --- | --- | --- | --- | --- | --- | --- | --- | --- | --- | --- | --- | --- |
| 1 | JFK | 1 | .26** | .21** | .45** | .38** | .34** | .24** | .29** | .31** | .31** | .28** | .41** | .13* | .22** | .07 | .26** | .22** | .35** | .21** | .24** |
| 2 | Scientology | .26** | 1 | .44** | .31** | .43** | .38** | .17** | .35** | .34** | .38** | .16** | .35** | .22** | .26** | .27** | .45** | .28** | .47** | .49** | .30** |
| 3 | Nuclear accidents UDSSR | .21** | .44** | 1 | .24** | .30** | .28** | .14* | .25** | .29** | 23** | .19** | .27** | .26** | .30** | .24** | .32** | .31** | .33** | .58** | .24** |
| 4 | 11^th^ September | .45** | .31** | .29** | 1 | .48** | .45** | .38** | .36** | .38** | .37** | .17** | .67** | .31** | .35** | .22** | .39** | .39** | .40** | .40** | .40** |
| 5 | Jewish families | .38** | .43** | .30** | .48** | 1 | .46** | .33** | .45** | .55** | .57** | .19** | .52** | .34** | .30** | .20** | .66** | .39** | .56** | .41** | .58** |
| 6 | Lady Di | .34** | .38** | .28** | .45** | .46** | 1 | .28** | .36** | .33** | .35** | .16** | .51** | .35** | .34** | .21** | .43** | .26** | .42** | .43** | .37** |
| 7 | Iraq invasion | .24** | .17** | .14* | .38** | .33** | .28** | 1 | .13* | .23** | .17** | .22** | .35** | .19** | .36** | .22** | .34** | .38** | .19** | .23** | .37** |
| 8 | Alien contact | .29** | .35** | .25** | .36** | .45** | .36** | .13** | 1 | .54** | .62** | .24** | .48** | .25** | .17** | .035 | .53** | 25** | .57** | .40** | .40** |
| 9 | Illuminati | .31** | .34** | .29** | .38** | .55** | .33** | .23** | .54** | 1 | .53** | .29** | .51** | .39** | .32** | .06 | .65** | .35** | .47** | .29** | .47** |
| 10 | Chemtrails | .31** | .38** | .23** | .37** | .57** | .35** | .17** | .62** | .53** | 1 | .20** | .54** | .23** | .21** | .02 | .58** | .27** | .58** | .39** | .51** |
| 11 | Marie Magdalene | .28** | .16** | .19** | .17** | 19** | .16** | .22** | .24** | .29** | .20** | 1 | .27** | .24** | 25** | .05 | 23** | .23** | .21** | .19** | .23** |
| 12 | World Trade Center | .41** | .35** | .27** | .67** | .52** | .51** | .35** | .48** | .51** | .54** | .27** | 1 | .35** | .36** | .12** | .50** | .32** | .45** | .38** | .49** |
| 13 | Human sacrifices | .14* | .22** | .26** | .31** | .34** | .35** | .19** | .25** | .39** | .23** | .24** | .35** | 1 | .35** | .31** | .39** | .35** | .26** | .35** | .31** |
| 14 | Stainless steel | .22** | .26** | .30** | .35** | .30** | .34** | .36** | .17** | .32** | .21** | .25** | .36** | .35** | 1 | .25** | .31** | .50** | .28** | .41** | .36** |
| 15 | Mind control | .07 | .27** | .24** | .22** | .20** | .21** | .22** | .04 | .06 | .02 | .05 | .12** | .31** | .25** | 1 | .23** | .25** | .16** | .22** | .19** |
| 16 | Freemasons | .26** | .45** | .32** | .39** | .66** | .43** | .34** | .53** | .65** | .58** | .23** | .50** | .39** | .31** | .23** | 1 | .40** | .53** | .44** | .53** |
| 17 | Pharmaceutical industry | .22** | .28** | .31** | .39** | .35** | .26** | .38** | .25** | .35** | .27** | .22** | .32** | .35** | .50** | .25** | .40** | 1 | .33** | .41** | .42** |
| 18 | Nazi UFOs | .35** | .47** | .33** | .40** | .56** | .42** | .19** | .57** | .47** | .58** | .21** | .45** | .26** | .28** | .16** | .53** | .33** | 1 | .48** | .48** |
| 19 | Nuclear accidents USA | .21** | .49** | .58** | .40** | .41** | .43** | .23** | .40** | .29** | .39** | .19** | .38** | .35** | .41** | .22** | .43** | .41** | .48** | 1 | .44** |
| 20 | Fate of Earth | .24** | .30** | .24** | .40** | .58** | .37** | .37** | .40** | .47** | .51** | .23** | .49** | .31** | .36** | .19** | .53** | .42** | .46** | .44 | 1 |

Notes: ^1^ See Table S2 and S3 for the specific conspiracy belief ^*^ p < .05 ^**^ p < .01^**^

**Table S30: Intercorrelations between individual conspiracy beliefs and populism**

|  | Conspiracy beliefs^1^ | *n* | Anti | Sov | Hom |
| --- | --- | --- | --- | --- | --- |
| 1 | JFK | 342 | .248, *p* < .001 | .542, *p* < .001 | .197, *p* < .001 |
| 2 | Scientology | 377 | .210, *p* < .001 | .207,  *p* < .001 | .181, *p* < .001 |
| 3 | Nuclear accidents UDSSR | 304 | .198, *p* < .001 | .129, *p* = .025 | .286, *p* < .001 |
| 4 | 11^th^ September | 453 | .306, *p* < .001 | .240, *p* < .001 | .082, *p* = .081 |
| 5 | Jewish families | 422 | .315, *p* < .001 | .265, *p* < .001 | .243, *p* < .001 |
| 6 | Lady Di | 435 | .281, *p* < .001 | .288, *p* < .001 | .181, *p* < .001 |
| 7 | Iraq invasion | 409 | .147, *p* = .003 | .149, *p* = .003 | .031, *p* = .536 |
| 8 | Alien contact | 449 | .235, *p* < .001 | .228, *p* < .001 | .137, *p* = .004 |
| 9 | Illuminati | 416 | .193, *p* < .001 | .279, *p* < .001 | .170, *p* < .001 |
| 10 | Chemtrails | 455 | .242, *p* < .001 | .211, *p* < .001 | .200, *p* < .001 |
| 11 | Marie Magdalene | 348 | .144, *p* = .007 | .169, *p* = .002 | .058, *p* = .278 |
| 12 | World Trade Center | 465 | .305, *p* < .001 | .319, *p* < .001 | .090, *p* = .057 |
| 13 | Human sacrifices | 391 | .247, *p* < .001 | .213, *p* < .001 | .173, *p* < .001 |
| 14 | Stainless steel | 357 | .363, *p* < .001 | .324, *p* < .001 | .077, *p* = .146 |
| 15 | Mind control | 452 | .097, *p* = .039 | .010, *p* = .838 | .103, *p* = .028 |
| 16 | Freemasons | 362 | .253, *p* < .001 | .239, *p* < .001 | .196, *p* < .001 |
| 17 | Pharmaceutical industry | 466 | .281, *p* < .001 | .235, *p* < .001 | .075, *p* = .107 |
| 18 | Nazi UFOs | 336 | .279, *p* < .001 | .231, *p* < .001 | .207, *p* < .001 |
| 19 | Nuclear accidents USA | 327 | .282, *p* < .001 | .190, *p* < .001 | .220, *p* < .001 |
| 20 | Fate of Earth | 430 | .391, *p* < .001 | .249, *p* < .001 | .072, *p* = .138 |

Notes: Anti = Anti-elitism Scale; Sov = Preference for unrestricted popular sovereignty Scale; Hom = Homogeneity and virtuousness of the people Scale ^1^ See Table S2 and S3 for the specific conspiracy belief.

**Table S31: Sociodemographic characteristics in the sample, including persons with diverse sex**

| *Sociodemographics* | *M/*(%)/*n* | *SD* |
| --- | --- | --- |
| Sex assigned at birth  female  male  diverse | 285 (58.40 %)  198 (40.57 %)  5 (1.03 %) |  |
| Age (in years) | 28.11 | 7.80 |
| Education level |  |  |
| No graduation | 1 (0.2 %) |  |
| Other degree ^1^ | 2 (0.4 %) |  |
| 9 school years ^2^ | 13 (2.7 %) |  |
| 10 school years ^3^ | 39 (8 %) |  |
| High school equivalent | 135 (27.7 %) |  |
| University degree (not specified) | 292 (59.8 %) |  |
| Ph.D. | 6 (1.2 %) |  |
| Affiliation to religious group |  |  |
| Roman-catholic | 175 (35.9 %) |  |
| Protestant | 129 (26.4 %) |  |
| Jewish | 3 (0.6 %) |  |
| Muslim | 10 (2 %) |  |
| Buddhist | 1 (0.2 %) |  |
| Jehovah’s witness | 1 (0.2 %) |  |
| Other Christian community | 7 (1.4 %) |  |
| Other | 6 (1.2 %) |  |
| No religion | 156 (32 %) |  |
| Religiosity | 1.84 | 0.84 |
| Political party to elect ^4^ |  |  |
| CDU/CSU ^5^ | 55 (11.3 %) |  |
| SPD ^6^ | 28 (5.7 %) |  |
| Green party | 199 (40.8 %) |  |
| FDP ^7^ | 58 (11.9 %) |  |
| AfD ^8^ | 7 (1.4 %) |  |
| Die Linke ^9^ | 57 (11.7 %) |  |
| Other | 41 (8.4 %) |  |
| I would not attend the election | 43 (8.8 %) |  |
| Diagnosis of mental disorder in the past ^10^ | Yes: 91 (18.6 %) |  |
| Depression | 61 (12.5 %) |  |
| Anxiety disorder | 26 (5.33 %) |  |
| Posttraumatic Stress Disorder | 11 (2.25 %) |  |
| Somatoform Disorder | 2 (0.4 %) |  |
| Eating disorder | 10 (2 %) |  |
| Obsessive-Compulsive disorder | 6 (1.2 %) |  |
|  |  |  |
| Endorsement of Conspiracy Beliefs Mean Score Scale (CB)  (*n* = 484) | 2.64 | 0.77 |
| Generalist Populist Attitudes mean score Scale (Gen) | 2.94 | 0.55 |
| Anti-Elitist Attitudes Scale (Anti) ^12^ | 3.31 | 0.73 |
| …Preference of Unrestricted Popular Sovereignty  Scale (Sov) | 3.25 | 0.88 |
| Belief in the Homogeneity and Virtuousness of the   People Scale (Hom) | 2.42 | 0.66 |
| Need for Cognition Scale (NC) | 5.04 | 0.95 |
| Faith in Intuition Scale (FI) | 4.10 | 0.81 |
|  |  |  |

Notes: ^1^ = another degree, e.g., special education degree; ^2^ = German Hauptschulabschluss (9 school years), ^3^ = German Realschulabschluss (10 school years), ^4^ = “If the election of the Bundestag would take place on Sunday, which party would you elect?”; ^5^ = Christliche Demokratische Union/Christliche Soziale Union (conservatives); ^6^ = Sozialdemokratische Partei Deutschland = middle-leftist party; ^7^ = Freie Demokratische Partei = conservative and liberal; ^8^ = Alternative für Deutschland = right-wing extremist, critical toward migration; ^9^ Die Linke = left-wing extremist party, former SED (Soziale Einheitspartei Deutschlands) from Eastern Germany; ^10^ = it was possible to choose several options.

**Table S32: Group differences in groups regarding sex assigned at birth in endorsement of conspiracy beliefs, populist attitudes, and analytical and intuitive thinking style, including persons with diverse sex assigned at birth**

|  | *Total group* | | *Male* | | *Female* | | *Diverse* | | *Group differences* |
| --- | --- | --- | --- | --- | --- | --- | --- | --- | --- |
|  | *n* | *M (SD)* | *n* | *M (SD)* | *n* | *M (SD)* | *n* | *M (SD)* | *Univariate ANOVA* |
| Endorsement of Conspiracy Beliefs (CB) | 484 | 2.64 (0.77) | 197 | 2.56 (0.83) | 282 | 2.68 (0.71) | 5 | 3.12 (0.79) | *F (*2,481) = 2.304, *p* = .101 |
| Generalist Populist Attitudes Scale (Gen) | 488 | 2.94 (0.55) | 198 | 2.87 (0.58) | 285 | 2.99 (0.53) | 5 | 2.72 (0.39) | *F (*2,485) = 2.632, *p* = .073 |
| Anti-Elitist attitudes Scale (Anti) | 488 | 3.31 (0.73) | 198 | 3.32 (0.78) | 285 | 3.31 (0.69) | 5 | 2.96 (0.78) | *F (*2,485) = .592, *p* = .554 |
| Preference of Unrestricted | 488 | 3.25 (0.88) | 198 | 3.13 (0.98) | 285 | 3.33 (0.79) | 5 | 3.15 (0.76) | *F (*2,485) = 3.188, *p* = .045 |
| Popular Sovereignty Scale (Sov) |  |  |  |  |  |  |  |  | ^1^female > male (*p* = .036) |
| Belief in the Homogeneity and Virtuousness of the People Scale (Hom) | 488 | 2.42 (0.66) | 198 | 2.34 (0.63) | 285 | 2.48 (0.68) | 5 | 2.23 (0.51) | *F (*2,485) = 2.922, *p* = .055 |
| Need for Cognition Scale (NC) | 488 | 5.04 (0.95) | 198 | 5.25 (1.01) | 285 | 4.91 (0.87) | 5 | 4.60 (1.07) | *F* (2,485) = 8.440, *p* < .001 |
|  |  |  |  |  |  |  |  |  | *^1^* male > female *(p* < .001*)* |
| Faith in Intuition Scale (FI) | 488 | 4.10 (0.81) | 198 | 3.95 (0.87) | 285 | 4.19 (0.76) | 5 | 4.29 (0.78) | *F (*2,485) = 5.275, *p* = .005 |
|  |  |  |  |  |  |  |  |  | ^1^ female > male *(p = .004)* |

**Table S33: Correlations between age, education level, and endorsement of conspiracy beliefs, populist attitudes, and analytical and intuitive thinking styles, including persons with diverse sex assigned at birth**

|  | Age (in years) | Education level |
| --- | --- | --- |
| Endorsemenf of Conspiracy Belief Scale (CB) | .112; *p* = .014 ^1^ | -.160, *p* < .001 ^1^ |
| Generalist Populist Attitudes Scale (Gen) | .034, *p* = .453 ^2^ | -.222 *p* < .001 ^2^ |
| Anti-Elitist Attitudes Scale (Anti) | .066, *p* = .148 ^2^ | -.181, *p* < .001 ^2^ |
| Preference of Unrestricted Popular | .019, p = .670 ^2^ | -.153, *p* < .001 ^2^ |
| Sovereignty Scale (Sov) |  |  |
| Belief in the Homogeneity and Virtuousness of the People Scale  (Hom) | -.006, *p* = .888 ^2^ | -.161, *p* < .001 ^2^ |
| Need for Cognition Scale (NC) | .117, *p* = .010 ^2^ | .078, *p* = .084 ^2^ |
| Faith in Intuition Scale (FI) | -.101, *p* = .026 ^2^ | -.099, *p* = .029 ^2^ |

Notes: M = Mean; SD = Standard deviation; ^1^ *n* = 484; ^2^ *n* = 488.

**References:**

Barri, M. A. (2019). A Simulation Showing the Role of Central Limit Theorem in Handling Non-Normal Distributions. *American Journal of Educational Research*, *7*(8), 591-598. <https://doi.org/https://doi.org/10.12691/education-7-8-8>.

Bartoschek, S. (2015). *Bekanntheit von und Zustimmung zu Verschwörungstheorien - eine empirische Grundlagenarbeit.* JMB Verlag.

Bauer, D. J., & Curran, P. J. (2005). Probing interactions in fixed and multilevel regression: inferential and graphical techniques. *Multivariate Behavioral Research*, *40*(3), 373-400. <https://doi.org/thhp://dx.doi.org/10.1207/s15327906mbr4003_5>

Brotherton, R., French, C. C., & Pickering, A. D. (2013). Measuring belief in conspiracy theories: the generic conspiracist beliefs scale. *Frontiers of Psychology*, *4*(279). <https://doi.org/https://doi.org/10.3389/fpsyg.2013.00279>

Cacioppo, J. T., & Petty, R. E. (1982). The Need for Cognition. *Journal of Personality and Social Psychology*, *42*(1), 116-131.

Epstein, S. (2003). Cognitive-experiential self-theory of personality. In T. Millon & M. J. Lerner (Eds.), *Handbook of psychology: Vol. 5. Personality and social psychology* (pp. 159–184). Wiley.

Epstein, S., Pacini, R., Denes-Raj, V., & Heier, H. (1996). Individual differences in intuitive-experiential and analytical-rational thinking styles. *Journal of Personality and Social Psychology*, *71*(2), 390-405. <https://doi.org/https://doi.org/10.1037//0022-3514.71.2.390>

Hayes, A. F., & Preacher, K. J. (2014). Statistical mediation analysis with a multicategorical independent variable. *British Journal of Mathematical and Statistical Psychology*, *67*, 451-470. <https://doi.org/10.1111/bmsp.12028>

Johnson, P. O., & Fay, L. C. (1950). The Johnson-Neyman technique, its theory and application. *Psychometrika*, *15*(4), 349–367. <https://doi.org/https://doi.org/10.1007/BF02288864>

Keller, J., Bohner, G., & Erb, H.-P. (2000). Intuitive und heuristische Urteilsbildung - verschiedene Prozesse? *Zeitschrift für Sozialpsychologie*, *31*(2), 87–101. <https://doi.org/https://doi.org/10.1024//0044-3514.31.2.87>

Mudde, C. (2004). The Populist Zeitgeist: Government and Opposition. *39*, *4*, 542–563. <https://doi.org/https://doi.org/10.1111/j.1477-7053.2004.00135.x>

Pytlik, N. (2016). *Voreiliges Schlussfolgern als Grundlage für Verschwörungsglauben (Bachelorarbeit)*. Philipps-Universität Marburg.

Schulz, A., Mueller, P., Schemer, C., Wirz, D. S., Wettstein, M., & Wirth, W. (2017). Measuring Populist Attitudes on Three Dimensions. *International Journal of Public Opinion Research*, *12*(8), 931. <https://doi.org/https://doi.org/10.1093/ijpor/edw037>

Weinberg, S. L., & Abramowitz, S. K. (2016). *Statistics using IBM SPSS: An Integrative Approach*. Cambridge University Press.

Woodward, T. S., Munz, M., LeClerc, C., & Lecomte, T. (2009). Change in delusions is associated with change in “jumping to conclusions. *Psychiatry Research*, *170*(2-3), 124–127. <https://doi.org/https://doi.org/10.1016/j.psychres.2008.10.020>
